# Ionic liquid-assisted seed genomic DNA extraction for advanced sequencing applications

**DOI:** 10.1186/s13007-025-01417-1

**Published:** 2025-07-16

**Authors:** Shashini De Silva, Philip C. Bentz, Cecilia Cagliero, Morgan R. Gostel, Gabriel Johnson, Jared L. Anderson

**Affiliations:** 1 Department of Chemistry, Iowa State University, Ames, IA 50011 USA; 2 HudsonAlpha Institute for Biotechnology, Huntsville, AL USA; 3 Dipartimento di Scienza e Tecnologia del Farmaco, Università di Torino, Turin, I-10125 Italy; 4 Botanical Research Institute of Texas, Fort Worth, TX 76107-3400 USA; 5 Smithsonian Institution, Suitland, MD 20746 USA

**Keywords:** Ionic liquids, High-molecular weight DNA, High-throughput sequencing, Plant breeding, Soybean, Seed DNA

## Abstract

**Background:**

Modern plant breeding strategies rely on the intensive use of advanced genomic tools to expedite the development of improved crop varieties. Genomic DNA extraction from crop seeds eliminates the need to grow plants in contrast to fresh leaf tissue; however, it can still be a bottleneck due to the presence of stored compounds and the complexity of the matrix. The interaction of environmentally benign choline-based ionic liquids (ILs) with DNA offers an innovative approach to enhance the quality of extracted DNA from seeds. While prior IL-based plant DNA extraction workflows have primarily supported polymerase chain reaction (PCR) and quantitative PCR-based applications, their suitability for high-throughput sequencing (HTS) remained largely unexplored. This study explores the efficacy of IL-assisted method for genomic DNA extraction from soybean (*Glycine max*) seeds, addressing the limited application of ILs in HTS.

**Results:**

The optimized DNA extraction method, utilizing 25% (w/v) choline formate, enabled the recovery of high-purity DNA with abundant fragment sizes > 20 kb, suitable for downstream applications including PCR, whole genome amplification (WGA), simple sequence repeat (SSR) amplification, and high-throughput Illumina sequencing. The IL-method was benchmarked against a silica-binding method using cetyltrimethylammonium bromide (CTAB) and sodium dodecyl sulfate (SDS) as lysis agents using a commercial plant DNA extraction kit in terms of DNA yield, purity, abundant DNA fragment size distribution, and integrity. In addition, DNA isolated from this method demonstrated successful PCR amplification of markers from both the nuclear and plastid genomes and yielded > 99% whole genome coverage with Illumina (PE150) sequencing reads.

**Conclusions:**

This is the first known instance of a whole genome sequence generated from DNA extracted with ILs. These findings mark a significant milestone in establishing ILs as promising alternatives to conventional methods for seed DNA extraction, with potential utility in third generation (long-read) sequencing experiments.

**Supplementary Information:**

The online version contains supplementary material available at 10.1186/s13007-025-01417-1.

## Background

Addressing challenges of global population growth requires continuous advancements in crop breeding to improve yield [1] and the development of more resilient and nutritious crops [2]. Modern breeding programs increasingly integrate genome information at a lower cost from next-generation sequencing (NGS) to enhance selection accuracy, thereby facilitating the rapid identification of agriculturally valuable genes that can be leveraged as tools in breeding [3]. HTS technologies such as Illumina, PacBio HiFi, and Oxford Nanopore Technology (ONT) have transformed plant breeding and facilitate comprehensive plant genome analysis, but require higher quantities of DNA for library preparation and sequencing. NGS requires often very stringent quality control, as the purity, yield and integrity of DNA dictate the quality of the final genome assembly [4, 5]. High-quality DNA suitable for long-read sequencing is typically high molecular weight (HMW) with minimal shearing possessing a 260 nm and 280 nm absorbance ratio between 1.8 and 2.0, and is free from contaminants such as protein, RNA, or polysaccharides [6]. 

The quality and quantity requirements for NGS analysis of nucleic acids originating from plants are often confined to extraction methods that use leaf materials [7–9]. Young, tender leaves are preferred for DNA extraction as the number of cells per unit area is greater; however, obtaining leaf material necessitates germination and cultivation, which can be both time-consuming and resource-intensive. Seeds may be considered suboptimal for DNA extraction due to their hard, recalcitrant structure and the presence of stored compounds such as proteins, polysaccharides, lipids, and secondary metabolites. However, recent advancements have demonstrated that DNA extraction directly from seeds is not only feasible but can also be advantageous for crop genomic analysis [3, 10]. Notably, extracting seed DNA eliminates the necessity for plant cultivation, significantly reducing the time, labor, and space required for plant growth. Additionally, seeds can be stored long-term at low temperatures or even at room temperature, providing a readily available source of material for DNA extraction, as needed for high throughput breeding programs and other studies.

While much attention has been placed on conventional protocols [11] and commercially available kits, extracting high-quality DNA from unconventional plant sources (e.g., seeds), remains challenging due to the presence of interfering components. Many of the reported studies on seed DNA extraction using conventional protocols and commercial kits employ either cetyltrimethylammonium bromide (CTAB) [12] or sodium dodecyl sulfate (SDS) [13, 14] for cell lysis. While these methods are effective, they often involve lengthy incubation periods, multiple processing steps, and hazardous reagents. In recent years, ionic liquids (ILs) have emerged as potential alternatives to conventional extraction reagents in DNA isolation workflows. ILs, also referred to as “designer solvents,” are composed of organic cations and a diverse array of anions that can be tuned to interact with biomolecules [15]. In particular, certain ILs have been remarkably efficient in achieving cell lysis and DNA extraction from plant matrices [16–18]. Among the diverse classes of ILs, choline-based ILs stand out as promising candidates for biomolecular extraction due to their cell lysis efficiency [19]; DNA extraction capabilities [20]; and favorable physico-chemical and environmental properties. Choline (2-hydroxyethyltrimethyl ammonium) chloride is a naturally occurring and water-soluble quaternary ammonium salt that is well recognized for its biocompatibility, low toxicity, and biodegradability. Choline-derived ILs have been shown to be safer alternatives to many conventional ILs that may pose environmental or human-health concerns [21]. In addition to DNA extraction, hydrated choline-ILs have been shown to exhibit long-term DNA stability for 6 months at room temperature [22]. 

The ability of choline-based ILs to interact with DNA provides an innovative approach to improve the quality of DNA extracted from seeds of important crop species. Previous IL-based plant DNA extraction workflows have been explored for quantitative PCR (qPCR)-based amplification techniques and have not been reported for HTS applications. Given the aforementioned advantages of choline-based ILs, this study aimed to explore IL-assisted isolation of HMW DNA from soybean (*Glycine max* [L.] Merr.) seeds, with the goal of generating a whole genome sequence for modern plant breeding applications. To systematically investigate the influence of choline-based ILs on HMW DNA extraction performance in terms of yield, purity and integrity, a series of ILs including choline formate, choline acetate, choline dodecanoate, and choline dodecyl sulfate were selected and studied. Choline formate, choline acetate, and choline dodecanoate were chosen to evaluate the effect of different alkyl chain lengths in the anion on DNA yield, quality, and integrity. Choline dodecanoate and choline dodecyl sulfate were chosen to explore the potential surfactant effect imparted by the anion whereas the “surfactant-like” choline bromide salt was included for its surfactant property originating from the cation. The optimized IL extraction method employing 25% (w/v) choline formate enabled the recovery of high-purity HMW DNA with abundant fragment sizes > 20 kb. DNA isolated using the IL method was sequenced using HTS to demonstrate and verify, for the first time, the applicability of IL-based DNA extraction methods to generate high-quality whole genome sequences from crop seeds.

## Results

### Optimization of IL-buffer conditions for genomic DNA extraction

**Fig. 1:**
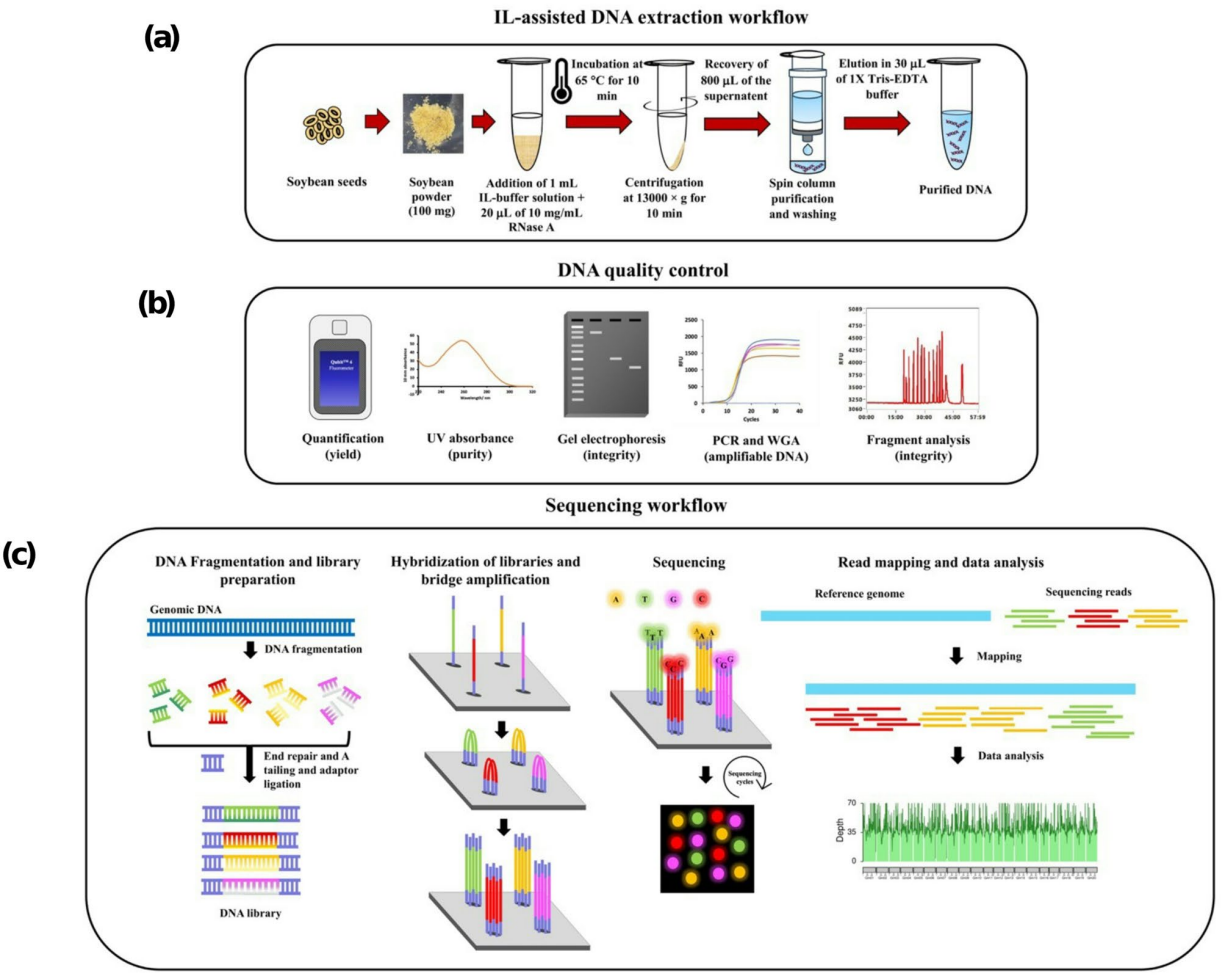
Schematic diagram illustrating **(a)** the IL-based extraction approach for isolating and purifying DNA from soybean seeds, **(b)** DNA quality control measures used in the study and **(c)** the standard sequencing workflow

The IL-based seed DNA extraction procedure employed in this study, along with the DNA quality control measures and the standard sequencing workflow, are illustrated in Fig. 1((a)-(c)), respectively. In the initial screening of ILs for DNA extraction, a 25% (w/v) IL solution was prepared in 1X TE (10 mM Tris-HCl, 0.1 mM EDTA, pH 8.0) buffer. Aqueous buffer solutions were used to decrease the IL viscosity and enhance the solubility of the biomolecules. Choline formate, choline acetate and choline dodecanoate were found to be completely soluble in the buffer while choline dodecyl sulfate and the surfactant-like choline bromide salt were completely insoluble at 25% (w/v) concentration. Although the “surfactant-like” choline bromide salt dissolved at elevated temperatures, the resulting solution was highly viscous, rendering it unsuitable for DNA extraction.

**Fig. 2:**
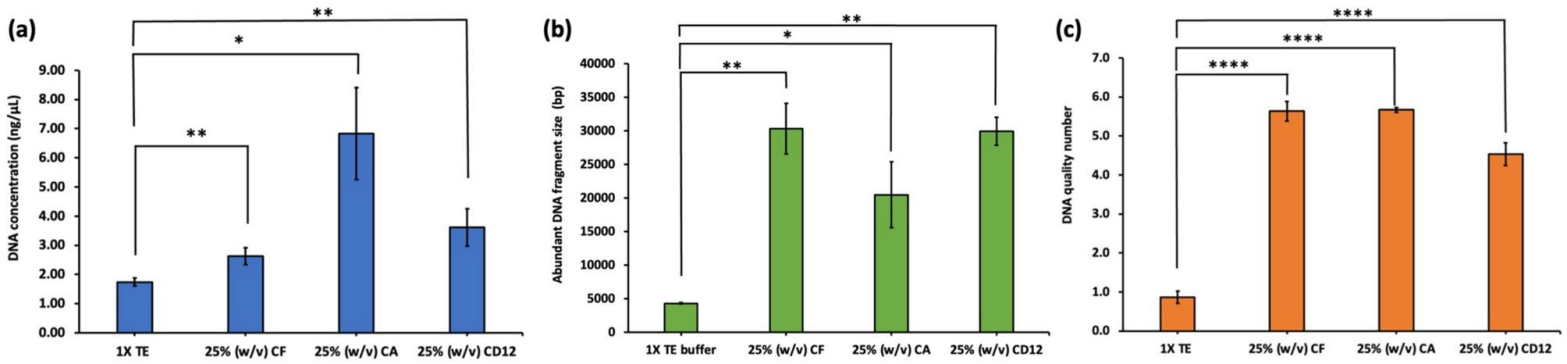
Influence of different choline-based ILs on the extraction of DNA from soybean seeds in terms of **(a)** DNA yield, **(b)** abundant fragment sizes, and **(c)** DNA integrity. Ground soybean powder of 100 mg weight was mixed with 1 mL of 25% (w/v) solution of IL in 1X TE buffer and 20 µL of 10 mg/mL RNase A, incubated at 65 °C for 10 min, centrifuged and the supernatant subjected to spin column purification. Statistical significance was determined using the Student’s t-test (*N* = 3). n.s. (no significance); *p < 0.05, **p < 0.01, ***p < 0.001, ****p < 0.0001. Error bars represent the standard deviation. (CF: choline formate; CA: choline acetate; CD12: choline dodecanoate)

As shown on Fig. 2, choline formate, choline acetate, and choline dodecanoate outperformed the standard TE buffer in terms of DNA yield, quality, and integrity. Among the tested ILs, choline formate, choline acetate, and choline dodecanoate successfully extracted DNA with moderate yields (Fig. 2(a)). DNA extracted using choline formate, choline acetate, and choline dodecanoate yielded abundant fragments greater than 10,000 bp, as shown in (Fig. 2(b)), whereas DNA extracted with neat 1X TE buffer exhibited abundant fragment sizes of 4310 ± 90 bp, indicating that the buffer alone yielded low molecular‑weight and highly fragmented DNA. These results indicate that choline-based ILs effectively extracted HMW DNA. The integrity of the extracted DNA was assessed using the DNA quality number (DQN), which is a quality metric developed by Agilent Technologies and used with fragment analyzer systems [23] to quantify the proportion of the total DNA concentration that exceeds a defined size threshold [23, 24]. Mulcahy et al. recommended using the ∼ 9 kb (= 9,416 bp) size marker as a working standard, as it is significantly longer than typical HTS reads, such as Illumina, and represents a suitable minimum threshold for long-read sequencing with current technologies [25]. In the current study, the threshold for DQN values was set at 10,000 bp. DQN values ranged from ≥ 4.0 to < 6.0 for IL-based extractions while the DQN for the neat 1X TE buffer was 0.9 ± 0.2, indicating poor DNA quality when using only TE buffer without the IL (Fig. 2(c)). Based on the successful results for HMW DNA extraction, subsequent experiments were conducted using choline formate, choline acetate, and choline dodecanoate.

**Fig. 3:**
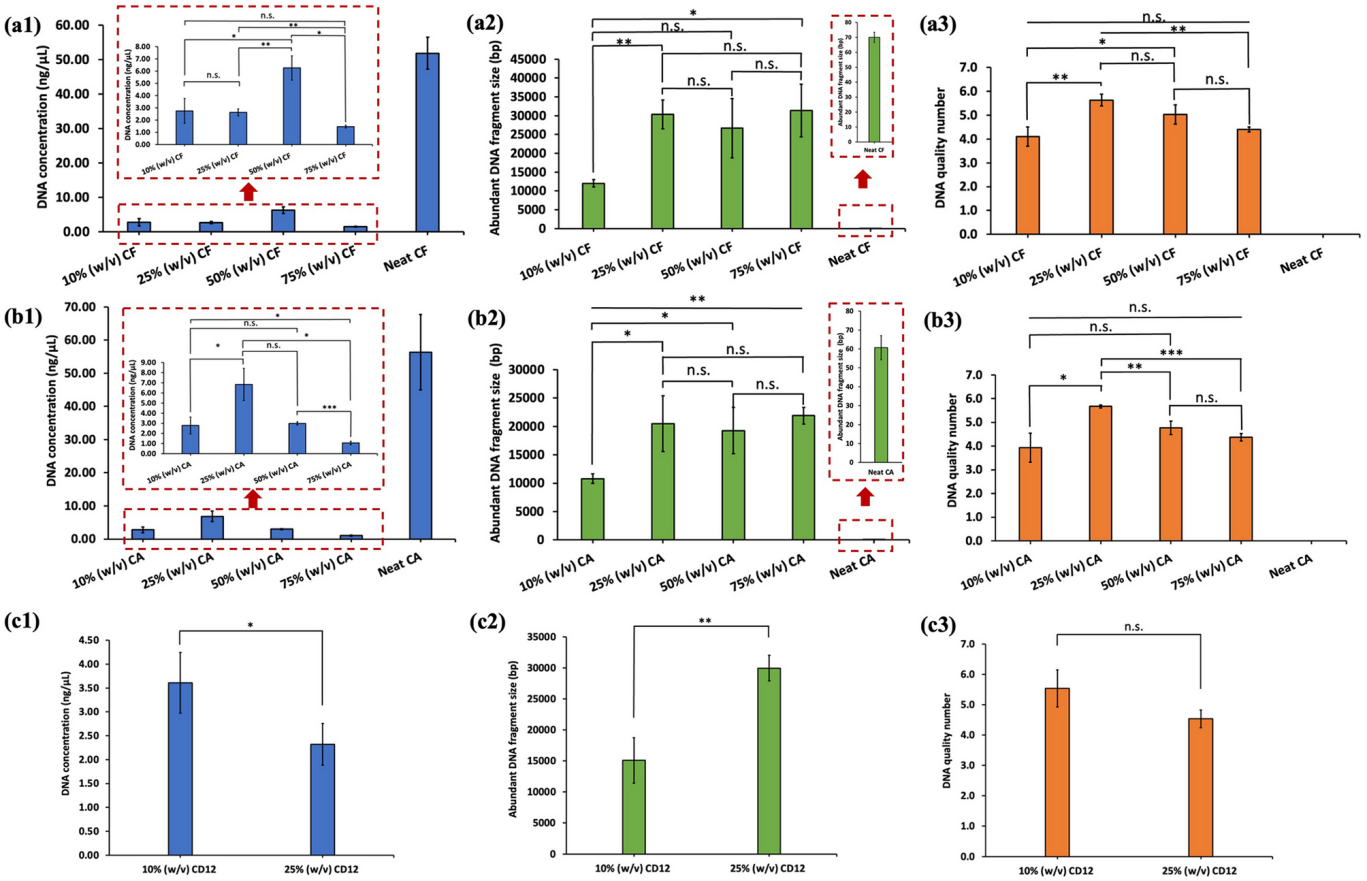
Influence of different concentrations of **(a)** choline formate (CF), **(b)** choline acetate (CA), and **(c)** choline dodecanoate (CD12) on the extraction of DNA from soybean seeds in terms of DNA yield (blue bars), abundant fragment sizes (green bars), and DNA integrity (orange bars). Ground soybean powder of 100 mg weight was mixed with 1 mL of IL-buffer solution (1X TE buffer) and 20 µL of 10 mg/mL RNase A, incubated at 65 °C for 10 min, centrifuged and the supernatant subjected to spin column purification. Statistical significance was determined using the Student’s t-test (*N* = 3). n.s. (no significance); *p < 0.05, **p < 0.01, ***p < 0.001, ****p < 0.0001. Error bars represent the standard deviation. Statistical comparisons between neat CF and neat CA with their respective aqueous mixtures for DNA yield and abundant DNA fragment size are included as Table S1

To evaluate the effect of IL concentration in the extraction buffer on DNA yield and integrity, the IL percentage (w/v) was systematically varied at 10%, 25%, 50%, 75% and 100% for choline formate, choline acetate, and choline dodecanoate. Choline formate and choline acetate were observed to be fully soluble in the buffer across all tested concentrations; however, choline dodecanoate was not soluble beyond 25% (w/v). A clear trend in DNA yield was not observed upon varying the IL concentration up to 75% (w/v) for the choline formate and choline acetate ILs. The DNA yield drastically increased for the neat (100% w/v) choline formate and choline acetate ILs (Fig. 3(a1) and 3(b1)). As shown in Fig. 3(a3), upon increasing the choline formate concentration in the buffer from 10 to 25%, the DQN increased from 4.10 ± 0.40 to 5.63 ± 0.25. The DQN for 50% (w/v) choline formate was found to be statistically similar (*p* > 0.05) to that of 25% (w/v) choline formate. Further increasing the IL concentration to 75% (w/v) decreased the DQN to 4.40 ± 0.10, indicating that higher choline formate concentrations may interfere with DNA quality. Although the DNA yield significantly increased upon using neat choline formate, DQN values of 0.00 were indicative of significant DNA degradation. The obtained DQN values were further supported by the distribution of abundant fragment sizes across a range of different IL concentrations. As shown in Fig. 3(a2), employing 10% (w/v) of choline formate more than doubled the abundant fragment length compared to that of the buffer alone (see Fig. 2(b)), suggesting some protective effects on DNA. A significant increase in the abundant fragment sizes to ∼ 30 kb upon using 25% (w/v) proved that much higher integrity DNA can be obtained. A 50% (w/v) choline formate solution also resulted in higher abundant fragment sizes of moderate variability. Although 75% (w/v) choline formate yielded large DNA fragments (∼ 36–38 kb), DQN values were lower than 5.0 indicating that a higher proportion of DNA had undergone degradation. Despite obtaining higher DNA concentration using neat choline formate, abundant fragment sizes were predominantly below 100 bp indicating near‑complete fragmentation. In the case of choline acetate, addition of the IL to the buffer progressively increased the abundant fragment lengths to 10,795 ± 835 bp at 10% (w/v), as shown in Fig. 3(b2), which was approximately 2-fold greater compared to that of neat 1X TE buffer and to 20,487 ± 4,899 bp at 25% (w/v) which is nearly 4-fold greater compared to that of 1X TE buffer alone. A statistical difference in the abundant DNA fragment sizes for 25–75% choline acetate was not observed. Beyond 75% (w/v), DNA integrity was observed to collapse in that neat choline acetate yielded almost no intact fragments. The DQN values increased up to 25% (w/v) and further increases in choline acetate concentration beyond this concentration resulted in decreased DQN values that approached zero for neat choline acetate. For the choline dodecanoate IL, an increase in concentration from 10% (w/v) to 25% (w/v) increased the abundant fragment length by approximately 2-fold indicative of longer DNA strands surviving shearing at the higher concentration level (Fig. 3(c2)). The similar DQN values with increasing choline dodecanoate concentration indicates that although longer abundant fragment lengths were achieved, the DNA fragment‑size distribution became more heterogeneous with a greater proportion of shorter fragments and an overall lowering of the integrity score.

To further investigate the presence of highly fragmented DNA for neat ILs, neat choline formate IL was incubated with soybean powder with and without RNase A treatment. Purified extracts that did not undergo RNase A treatment exhibited higher yields compared to those treated with RNase A (Figure S1(a)), indicating that in the absence of RNase A, total nucleic acids were extracted whereas with RNase A treatment, only DNA was extracted. Electropherograms for purified DNA that were initially not treated with RNase A demonstrated a considerable proportion of short fragments (up to 600 bp) compared to those treated with RNase A (Figure S1(b)). Even after RNase A treatment, the short fragments persisted and were not likely to be RNA. Furthermore, UV absorbance spectra confirmed a characteristic 260 nm peak, indicative of nucleic acids (Figure S1(c)). The initial RNase A treatment removed RNA contamination, confirming that the detected nucleic acids were primarily DNA. To assess whether the first-eluted, purified DNA fraction from the spin column consisted of only short fragments and that longer fragments remained bound to the spin column, a second elution was performed. However, the second elution also predominantly comprised short fragments. Additionally, the IL solution flowthrough from the initial binding step was reapplied to the spin column followed by the washing steps. Despite these efforts, the final eluted DNA remained highly fragmented (Figure S2).

To further investigate if DNA degradation takes place in the presence of neat ILs during extraction or from spin column purification, control experiments were conducted by spiking 100 µL lambda DNA in 700 µL of the choline formate IL followed by spin column purification. Upon spiking DNA with neat IL followed by spin column purification, a shift in the abundant fragment sizes were observed; however, unlike the observations with soybean DNA extracted by neat ILs, fragments shorter than 100 bp were not detected (Figure S3). This confirms that spin column purification itself does not result in fragmented DNA. Similarly, lambda DNA was incubated with the IL followed by gel electrophoresis on the IL extracts to assess the role of IL in DNA degradation. However, shorter fragments less than 100 bp were not observed for DNA spiked in ILs (data not shown). Although higher concentrations of ILs such as neat choline formate and choline acetate resulted in significantly increased yields for soybean seed DNA, these conditions led to substantial DNA fragmentation (Figs. 3(a2) and 3(b2)). Nanodrop UV absorbance spectra (Figure S4) confirmed that the DNA extracted using neat ILs exhibited acceptable purity; however, the integrity of the recovered DNA was compromised. Considering both the DQN value and the abundant fragment sizes, 25% (w/v) choline formate was chosen as the optimum IL composition for further optimization of the buffer conditions.

To evaluate the possibility of DNases from the plant matrix affecting overall DNA integrity, 20 µL of 10 mg/mL proteinase K was added to the supernatant of the 25% (w/v) choline formate extracts, as well as the 1X TE extracts, following a 10 min centrifugation step and incubated for 10 min at 55 °C. After subsequent washing and elution steps, the purified DNA from both 25% (w/v) choline formate extracts and 1X TE extracts did not improve DQN values indicating that the presence of nucleases may not be the culprit for slight DNA degradation observed in the electropherograms (Figures S5(a) and S5(b)). Abundant fragment sizes resulting from 25% (w/v) choline formate extracts in the presence of proteinase K were also superior compared to the 1X TE buffer extracts, further confirming the role of IL in HMW DNA extraction.

The effect of EDTA within the extraction buffer on the integrity of purified DNA was also assessed using concentrations of 0.1 mM, 1 mM, 10 mM, and 50 mM by keeping the IL and Tris-HCl concentration constant at 25% (w/v) and 10 mM, respectively. No significant changes in the integrity of DNA in terms of DQN values were observed upon varying the EDTA concentration (Figure S6(a)). The abundant fragment lengths for the 0.1 mM, 1 mM, and 50 mM solutions were statistically similar whereas the abundant fragment lengths for 10 mM buffer was lower compared to that of 0.1 mM EDTA (*p* < 0.05). Despite this, all solutions generated fragment lengths greater than 18,000 bp (Figure S6(b)). The buffer solution containing 0.1 mM EDTA was subsequently selected for further experiments. Although 10 mM of Tris–HCl (pH ≈ 8.0) along with EDTA was used to dilute the IL and maintain the pH of the solution, it was observed that 25% (w/v) choline formate resulted in acidic conditions in the overall solution and a pH value of approximately 5. Adjusting the pH of 25% (w/v) choline formate in 1X TE to a value of 8.0 using sodium hydroxide did not enhance the DQN and produced results comparable to the pH unadjusted buffer (Figure S7). To investigate the effect of pH, the concentration of Tris-HCl in the buffer was increased to keep the IL concentration constant at 25% (w/v) and EDTA concentration at 0.1 mM. As shown in Figures S8(a) and S8(b), increasing the Tris-HCl concentration to 500 mM resulted in an increased buffer pH value of approximately 8.0 and a slight improvement in DQN values (compared to that of 10 mM), respectively. At all Tris-HCl concentrations tested with the IL, the abundant fragments were greater than 20,000 bp and no statistically significant differences were observed, suggesting that the extraction of large DNA fragments was independent of Tris-HCl concentration (Figure S8(c)). For subsequent experiments, 25% (w/v) choline formate in 500 mM Tris-HCl and 0.1 mM EDTA was chosen as the optimal buffer for extraction.

### Versatility of the IL extraction method across different soybean varieties in comparison to CTAB and SDS lysis methods

**Fig. 4:**
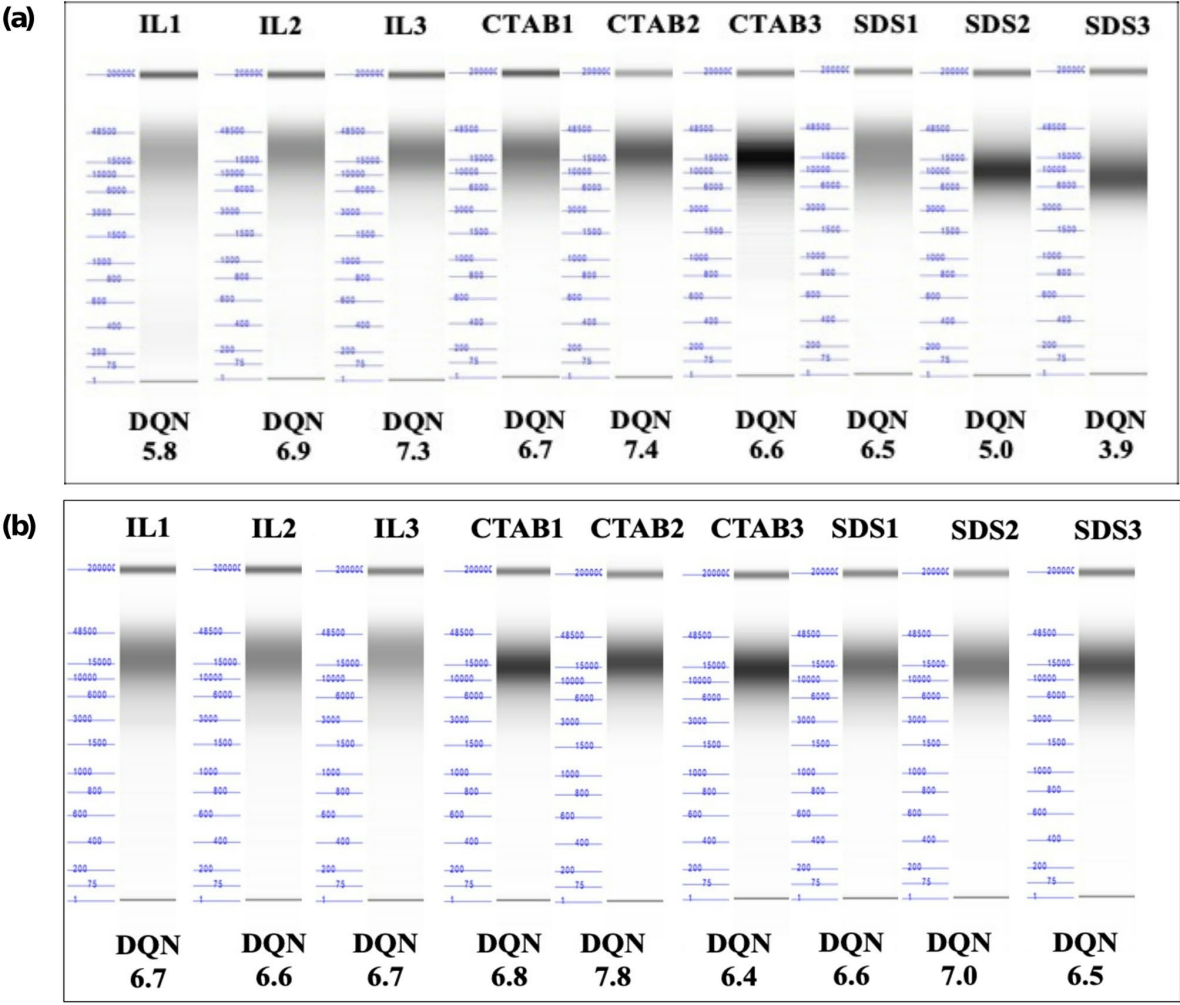
Agilent fragment analysis for DNA extracted from **(a)** soybean seed variety 1 and **(b)** Williams82 seeds by the IL, CTAB, and SDS methods. Lower marker: 1 bp, upper marker: 200,000 bp

A comparison of the IL extraction method for two soybean varieties was evaluated. DNA yield, integrity, and the most abundant fragment sizes were comparable for both soybean seed varieties indicating that the IL method is applicable to different soybean varieties (Figure S9). Additionally, both varieties were subjected to CTAB and SDS-based lysis followed by spin column purification and the results were compared with the IL-based extraction method in terms of DNA yield, purity, integrity, and abundant fragment size (Figures S10(a1-a3) and S10(b1-b3)). While the DNA yield from the IL-based extraction was lower than that from the CTAB and SDS-based methods, likely due to the surfactant properties of CTAB and SDS, DNA integrity remained consistent across all methods for both varieties. This was further supported by the DQN values, which showed no statistically significant differences among the three extraction methods (*p* > 0.05), as shown by Figures S10(a3) and S10(b3)). Although the average of the abundant fragment sizes for seed variety 1 was not statistically significant across the three methods, abundant fragment sizes obtained using SDS and CTAB methods showed greater variability, ranging from 8,237 bp to 23,731 bp and 14,600 bp to 23,528 bp, respectively (Figure S10(a2)). In contrast, the IL-based method consistently yielded abundant fragments exceeding 20,000 bp across all three replicates, suggesting enhanced repeatability. For Williams 82, the abundant fragment sizes were not statistically different for CTAB and IL-based methods. However, the SDS method yielded average abundant fragment sizes below 20,000 bp. The observed DNA integrity for all three methods was similar (Figure S10(b3)). Gel images for the DNA from each method are shown in Fig. 4.

**Fig. 5:**
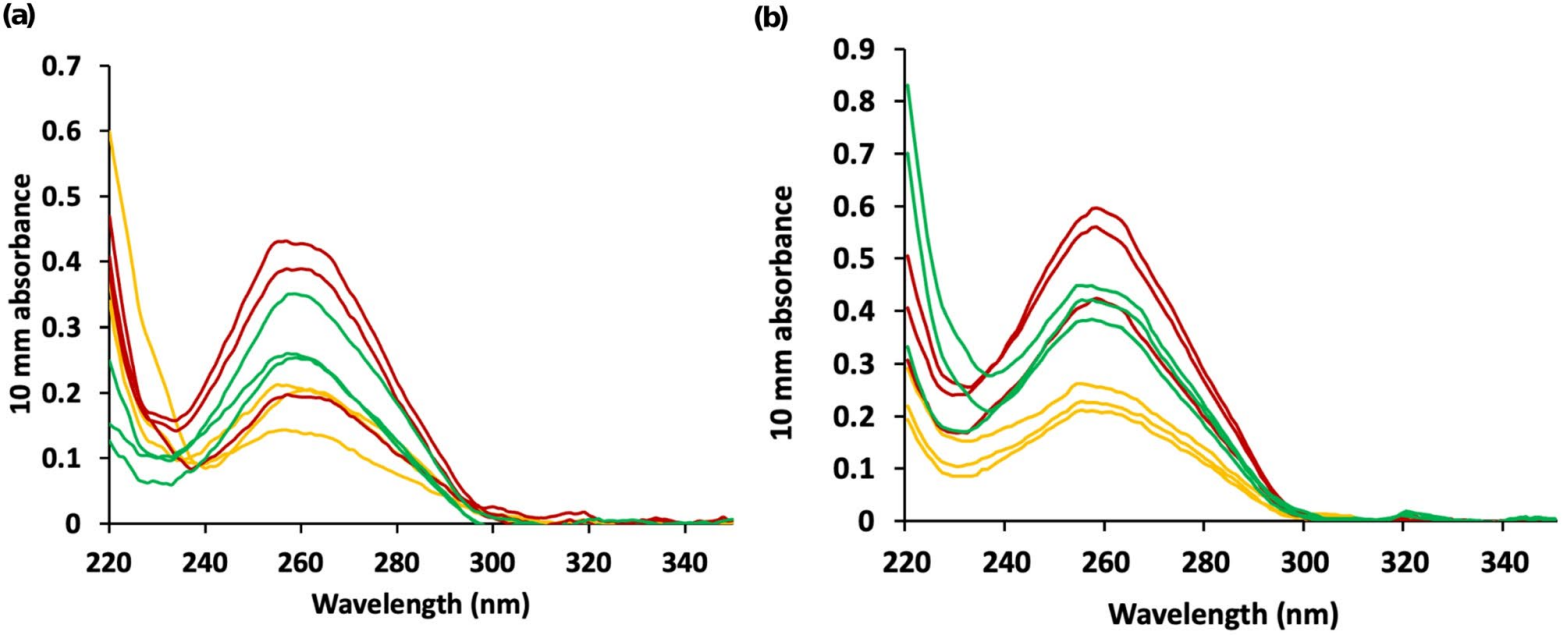
NanoDrop UV absorption spectra for DNA extracted from **(a)** soybean seed variety 1 and **(b)** Williams82 seeds by the IL (yellow), CTAB (red), and SDS (green) methods

The purity of each extracted DNA sample was verified by spectrophotometry using NanoDrop as DNA and proteins have characteristic UV absorption peaks at 260 nm and 280 nm, respectively. NanoDrop results confirmed the presence of DNA in both soybean seed samples extracted using the IL method, as observed from their distinct UV absorption at 260 nm (Fig. 5(a) and 5(b)). The relatively low UV absorbance at 260 nm for the IL method compared to that of CTAB and SDS methods can be attributed to lower concentrations of DNA extracted by the IL method, consistent with the Beer-Lambert law relating absorbance to concentration. An absorbance ratio at 260 nm to 280 nm (260/280 ratio) of approximately 1.8 is acceptable for pure DNA [24]. The average 260/280 ratio for DNA extracted using the IL method was approximately 1.8, suggesting minimal protein contamination despite the protein-rich composition of soybean seeds (Table 1). In contrast, the CTAB and SDS methods yielded average 260/280 ratios exceeding 1.8. The 260/230 ratio serves as a secondary indicator of DNA purity, with an expected range of 2.0 to 2.2. Among the tested samples, only the CTAB and IL methods applied to soybean variety 1 and Williams 82 samples, respectively, resulted in 260/230 ratios within the acceptable range. The deviation observed in other samples may be attributed to contamination by polysaccharides from the plant matrix or the presence of guanidine hydrochloride or salts introduced during the DNA binding and washing steps.

**Table 1:** Summary of NanoDrop UV absorbance ratios for DNA extracted by the three different extraction methods examined in this study

Sample	Extraction method	Absorbance 260/280 nm	Absorbance 260/230 nm
Soybean seeds (variety 1)	IL method	1.79 ± 0.16	1.18 ± 0.49
	CTAB method	1.96 ± 0.11	2.15 ± 0.69
	SDS method	2.02 ± 0.14	3.32 ± 0.72
Soybean seeds (Williams 82)	IL method	1.89 ± 0.04	2.07 ± 0.41
	CTAB method	2.12 ± 0.03	2.33 ± 0.21
	SDS method	2.05 ± 0.05	1.69 ± 0.62

### Simple sequence repeat (SSR) amplification of extracted DNA

To assess if the extracted DNA is suitable for molecular marker amplification, genotyping was performed by real-time amplification of simple sequence repeat (SSR) markers using DNA extracted by the IL method, as well as the CTAB and SDS based methods serving as templates for amplification. Successful amplification was achieved for all 4 markers and no difference in peaks from the melting curve for each SSR marker was observed for amplified DNA from all three methods (Figures S11 (a1)-(d1)). The SSR amplification products were further visualized by gel electrophoresis (Figures S11 (a2)-(d2)). Non-specific amplification for the no-template control (NTC) reaction was observed for the Satt157s marker; however, this was not due to contamination or issues with any of the DNA extraction methods. The NTC displayed a distinct band with a different size and melting temperature compared to the positive samples. Despite this, product bands from the positive samples were distinct and clearly distinguishable from the NTC, ensuring reliable interpretation of the results.

### Real-time amplification of nuclear and plastid DNA

To further evaluate the suitability of the IL-based extraction method for downstream amplification techniques, real-time amplification of nuclear and plastid DNA regions was performed. Successful amplification of both nuclear and plastid markers was achieved using DNA extracted by the IL method, with melt peaks of the resulting amplicons comparable to those obtained using DNA extracted by the CTAB and SDS-based methods (Figure S12 (a1)-(b1)). This result indicates that the IL-method can extract both nuclear as well as plastid DNA. Additionally, the specificity of plastid DNA amplification was assessed using universal *rbcLa* primers. Single melt peaks and distinct product bands visualized on agarose gels (Figure S12) confirmed successful and specific amplification, further demonstrating that DNA extracted by the IL method is of high quality and comparable to DNA extracted using conventional methods. These results indicate that the IL-based extraction method is a reliable alternative for obtaining both nuclear and plastid DNA suitable for downstream molecular applications.

### Whole genome amplification (WGA) of extracted DNA

To test the efficiency of WGA as a quality control metric on the DNA extracted from Williams 82 soybean seeds, WGA was performed using 1 µL of DNA with a concentration of 2.00 ± 0.5 ng/µL, followed by evaluation of the yield and size of the amplified fragments. WGA was observed to increase total DNA by one to two orders of magnitude across the three methods, demonstrating its effectiveness at converting minute template amounts to higher yields. The highest WGA yield was shown by the DNA extracted by the CTAB-based method while both IL- and SDS-based methods offered similar yields, as shown in Table S2. The percentage increase in DNA concentration by WGA for the CTAB method was 5456 ± 1251% and that of the IL and SDS methods were 928 ± 274% and 1196 ± 581%, respectively. The CTAB extraction approach delivered the least‑inhibited template DNA (likely higher purity), thereby maximizing polymerase efficiency during WGA. IL and SDS methods produced less WGA product yields possibly due to residual inhibitors; therefore, they may be preferable only when moderate amplification is sufficient. Although IL-method yielded DNA with A260/280 and A260/230 ratios of 1.89 ± 0.04 and 2.07 ± 0.41 indicative of low contaminant levels (Table 1), low WGA product yields suggests the possible presence of trace levels of inhibitors in IL-extracted DNA that are not detectable by UV absorbance but may interfere with sensitive enzymatic processes such as WGA. Despite the higher yields, none of the three methods yielded abundant fragments (> 10,000 bp) for the amplified DNA. DQN values of less than 3.0 were obtained indicating that the majority of the amplified fragments were less than 10,000 bp.

### DNA quality analysis using whole genome sequencing (WGS)

**Fig. 6:**
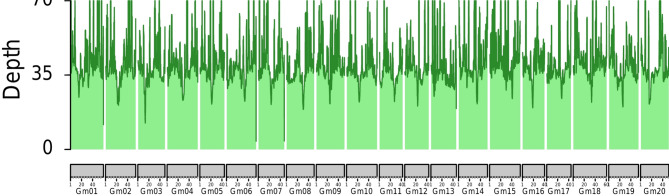
Illumina read mapping depth across all pseudo-chromosome assemblies from the soybean reference Wm82.a6.v1 [34]. Depth was summarized in 1 Mb sliding windows (0.9 Mb overlap) and plotted with karyoploteR v1.16.0 [38]. A maximum cutoff of 70x depth was applied for illustration purposes

HTS experiments performed on DNA extracted by the IL-method resulted in virtually perfect genome coverage based on Illumina read mapping analysis. An average of ∼ 99.8% of all bases per chromosome were covered by reads in the unfiltered dataset, whereas ∼ 94.8% of such was observed in the stringently filtered dataset (Table S3), which are typical results for HTS experiments. Further, sequencing results were relatively uniform across the genome (i.e., no signs of sampling bias from select regions of the genome), as mean sequencing depth was around 44.1x (Fig. 6) and 37.9x (Figure S13) across all chromosomes in the unfiltered and filtered datasets, respectively. Mean base quality scores were 39.6 across all Illumina sequencing reads, which equate to being essentially error free (> 99.9% accurate). Mean base quality scores are on a 1–40 scale and > 30 quality scores suggest bases are > 99.9% accurate.

### Evaluation of the IL method for DNA extraction from maize seeds in comparison to CTAB and SDS lysis methods

In addition to soybean seeds, DNA was extracted from maize seeds using IL, CTAB, and SDS-based methods followed by spin column purification, and evaluated in terms of yield, purity, and fragment size distribution. The IL (16.1 ± 2.86 ng/µL) and SDS methods (19.9 ± 0.46 ng/µL) produced similar yields (*p* > 0.05), as shown in Table S4, while the CTAB method yielded significantly lower amounts of DNA (1.57 ± 0.07 ng/µL). NanoDrop results indicated characteristic UV absorption of DNA for both IL and SDS methods (Figure S14). In contrast, the CTAB method yielded DNA at concentrations below the detection limit, resulting in the absence of characteristic DNA absorbance peaks (data not shown). Both the IL and SDS methods yielded DNA purities, as indicated by A260/280 ratios near 2.0 and A260/230 ratios between 1.6 and 1.7, whereas the CTAB method exhibited poor purity (A260/280: 0.53 ± 0.58). The quality and size distribution of DNA extracted from maize seeds using the IL, CTAB and SDS methods were further characterized by fragment analyzer. Both the IL and SDS methods yielded DNA with a broad size distribution, indicative of fragmented genomic DNA. Within this broad distribution, a prominent primary peak appeared around 2.5–2.8 kb and a second, broader peak was also observed at HMW region (Figure S15(a) and S15(c)). The presence of the overall smear rather than distinct HMW bands indicated that the extracted genomic DNA from maize seeds was fragmented. The CTAB method produced DNA of very low quality with a broad smear (Figure S15(b)). Major peaks, similar to those seen in the IL and SDS methods, were largely absent. Instead, the profiles often showed a diffuse, low-level signal across a wide range of sizes, indicative of severe DNA degradation or high levels of co-purified contaminants that interfered with accurate DNA detection and sizing.

## Discussion

This study reports the systematic development and successful application of IL-assisted DNA extraction as an effective and scalable method for isolating HMW DNA from plant seeds, suitable for high-throughput, WGS on the Illumina platform. Although not tested here, the IL-based extractions yielded longer DNA fragments that are in the range expected for third generation, long-read sequencing technologies (i.e., 20–30 kb). While previous IL- and magnetic IL-based extraction approaches for plant matrices have primarily focused on amplification-based downstream applications such as PCR and qPCR [16, 26], this study explored the potential of ILs as viable alternatives to conventional extraction methods such as CTAB- and SDS-based protocols for HTS applications. By achieving both high DNA integrity and sufficient purity, this IL-based method represents a significant advancement in the pursuit of efficient and scalable extraction techniques involving environmentally benign solvents for modern genomics workflows.

Among the ILs screened, choline formate, choline acetate, and choline dodecanoate emerged as the most effective for extracting DNA. Choline dodecyl sulfate and the surfactant-like choline bromide salt were insoluble at the tested concentration of 25% (w/v), and even upon solubilization at elevated temperatures, produced highly viscous solutions that were unsuitable for DNA extraction. These findings highlight that both the solubility and physico-chemical compatibility of the IL with biomolecular components are critical for successful extraction.

During screening of choline formate, choline acetate and choline dodecanoate ILs, 25% (w/v) choline formate in 1X TE buffer yielded DNA with abundant fragment sizes approaching 30 kb and DQN values ≥ 5.5, indicating moderate DNA integrity. Increasing the IL concentration beyond this point enhanced DNA yield drastically for neat ILs but significantly compromised integrity, as evidenced by lower DQN values and extensive fragmentation, likely due to extreme ionic strength or acidic pH effects on the DNA structure. This result highlights the importance of optimizing both IL concentration and buffer conditions to obtain the desired quality as well as the yield. It is also important to note that when developing an extraction method, obtaining higher yields with greater purity, as shown by UV absorption spectra, may not necessarily indicate higher integrity for DNA, as in the case of the neat choline formate and choline acetate ILs.

The effect of buffer composition, including Tris-HCl and EDTA concentration, on the extracted DNA was systematically evaluated. Increasing the concentration of Tris-HCl to 500 mM restored the overall buffer pH to ∼ 8.0 with an appreciable improvement in the integrity of DNA, while varying the EDTA concentration had minimal impact. This suggests that pH stabilization, rather than chelation of divalent ions alone, is critical for maintaining DNA stability during extractions in IL-aqueous buffer systems.

It was also observed that the IL-based method resulted in lower total DNA yields compared with commercial kit extraction methods (with CTAB and SDS lysis protocols), likely due to the lack of strong surfactant properties, yet the extracted DNA exhibited comparable integrity and purity. DQN values and fragment size distributions for the IL method were consistent across the tested soybean varieties, highlighting the robustness of the method. CTAB and SDS methods demonstrated higher variability in fragment sizes between replicates, while the IL method consistently yielded abundant fragments (> 20 kb), making it well-suited for both short- and long-read sequencing applications. Furthermore, the IL method was successful in extracting HMW DNA from other soybean varieties.

Molecular marker-assisted selection is a valuable tool in crop improvement, as numerous markers have been identified for soybean that are linked to disease resistance genes and important agronomic traits [27]. To evaluate the suitability of DNA extracted using the IL-based method for such applications, amplification was performed using SSR markers. The SSR amplification products were comparable to those obtained with CTAB and SDS methods. Similarly, amplification was performed targeting the nuclear and plastid regions of the extracted DNA. Successful PCR amplification revealed that the IL method extracts DNA from both the nuclear and plastid genomes. WGA has been identified as a valuable tool in recovering high yields of DNA when the sample amount or quantity of DNA for genomic analysis is limited. In contrast to PCR, which selectively amplifies specific DNA sequences, WGA aims to amplify the entire genome thereby minimizing amplification bias [28]. Although early WGA methods were PCR-based, the Qiagen REPLI-g Ultrafast Mini Kit used in this study employs multiple displacement amplification, which utilizes Phi29 DNA polymerase for high-fidelity WGA under isothermal conditions. WGA can be very useful in NGS, single-nucleotide polymorphism genotyping, clinical analysis, etc. However, in the present study, the efficiency of WGA was explored as a quality control metric on the extracted DNA from the three methods used in the study. WGA was effective across all three extraction methods, though the CTAB method generated the highest yield, likely due to reduced inhibitor content. While the IL method has demonstrated reduced carryover of common polysaccharide or protein contaminants, certain minor co-extracted metabolites or salts introduced during the DNA binding and washing steps may remain in the eluate and interfere with enzymatic reactions such as WGA, even if not at levels that significantly impact PCR or sequencing. Further analysis is necessary to identify and eliminate such residuals in future optimization of the IL extraction protocol. Nevertheless, DNA extracted by the IL-method produced sufficient WGA yield comparable to that of the SDS method. DNA from all three methods that were subjected WGA had abundant fragment lengths greater than 10 kb; however, the WGA products were less than 10 kb indicating preferential amplification of short DNA fragments.

This study represented the first WGS experiment performed with IL-based DNA extraction. Read mapping analysis yielded near-perfect coverage across the entire reference genome sequence (∼ 99.8% of all bases for each chromosome) and with > 40x depth.

Building upon these successful findings with soybean, the applicability and performance of the IL method for DNA extraction from maize seeds, a species known for its distinct compositional characteristics and often challenging DNA isolation, was evaluated. The IL and SDS methods provided the highest DNA yields from maize seeds with acceptable purities compared to the CTAB method however, the extracts still largely comprised fragmented DNA. This contrasts with the highly effective HMW DNA extraction observed with the IL method for soybean varieties. The CTAB method proved to be largely ineffective for maize seeds under the tested conditions, yielding very low DNA concentrations, poor purity ratios indicative of severe contamination, and highly degraded DNA profiles.

A notable discrepancy in the performance of the IL method between soybean and maize seeds was observed. While the IL method proved to be effective at extracting HMW DNA and providing moderate yields from soybean, its application to maize seeds, under conditions previously optimized for soybean, did not yield comparable results in terms of HMW DNA integrity. This suggests that the unique biochemical composition of maize seeds, which includes different levels of polysaccharides, starches, and other secondary metabolites, may necessitate specific modifications to the IL-based protocol. Specifically, tougher seed coats and distinct cellular matrix of maize likely pose different challenges for achieving efficient cell lysis and removal of contaminants that were not fully addressed by the existing IL protocol, which was optimized for soybean. Therefore, the current data for IL-based DNA extraction from maize seeds should be considered preliminary. Further optimization, including adjustments to lysis conditions, buffer compositions, and purification steps, is crucial to enhance DNA integrity and preserve HMW DNA from maize seeds, thereby unlocking the full potential of the IL method for various genomic applications in this important crop species.

## Conclusions

This study presents the successful development and application of choline-based ILs for HMW DNA extraction from soybean seeds that is compatible with downstream genomic analyses, including WGS. The aqueous buffer solution containing 25% (w/v) choline formate IL provided the optimal purity and integrity of DNA, consistently producing abundant fragment sizes exceeding 20 kb. Although this IL-based method yielded lower DNA quantities compared to commercial kit extraction methods utilizing CTAB and SDS lysis protocols, it provided comparable or superior DNA integrity and purity. The isolated DNA was successfully applied in a range of molecular biology applications, including PCR amplification of nuclear and plastid DNA markers, SSR marker amplification, and WGA, demonstrating the broad utility of the method. Furthermore, the IL-based DNA extraction method and the commercial kit extraction methods that involved CTAB and SDS lysis followed by spin column purification, required approximately 33 min from lysis to final DNA elution step. The conventional CTAB and SDS-based protocols typically require ∼ 1 h of extended lysis steps followed by chloroform: isoamyl alcohol purification, alcohol precipitation and DNA reconstitution. This time-saving advantage, combined with the ability of the method to yield DNA of sufficient purity and integrity, demonstrates its potential for high-throughput genomic workflows.

To the best our knowledge, this is the first demonstration of plant DNA extracted via an IL-based method being successfully utilized for Illumina HTS. Previous studies, including those employing miniaturized vortex-assisted matrix solid-phase dispersion with ILs have demonstrated compatibility with Sanger sequencing, but not with more demanding preparation methods such as those for Illumina sequencing. The successful generation of high-quality DNA-sequence data with relatively uniform and comprehensive sequence sampling (i.e., representing the entire genome equally) underscores the robustness of the IL method and represents an important step forward in integrating extraction workflows employing ILs with modern genomics. These findings mark an important new development for IL applications for the isolation of nucleic acids and their utilization in downstream genomic analyses – notably that IL-based DNA extraction is an affordable and scalable alternative to current methods that generates comparatively-high quality DNA. This study is the first of its kind to implement choline-based ILs for DNA extraction leading to the generation of a whole genome sequence and future efforts should prioritize optimization of choline-based IL extraction products for an expanded set of HTS applications, including so-called third-generation, long read platforms such as those developed by Oxford Nanopore Technologies and PacBio. Advances in IL-based nucleic acid isolation, particularly those presented in this work have shown great potential to meet the growing demands necessary to achieve and carry out large-scale genome sequencing efforts, such as the African BioGenome Project [29]; Darwin Tree of Life Project [30]; and the Earth BioGenome Project [31] that will require affordable, high-volume DNA extraction methods like that we have presented in this study.

While the IL method was highly effective for soybean, its optimal application to diverse plant seed varieties requires targeted protocol refinements. Future work will therefore focus on optimizing IL-based extraction to achieve robust HMW DNA yields from different seed varieties, further broadening its utility in plant genomics.

## Methods

### Chemicals and materials

Soybean (*Glycine max* (L.) Merr.) seeds were used as experimental material for optimization of HMW DNA extraction. Williams 82 seeds (Accession PI 518671) obtained from the U.S. National Plant Germplasm System were used for sequencing due to the availability of the reference genome. Maize (*Zea mays* subsp. *mays*, accession PI 550473) seeds were obtained from the U.S. National Plant Germplasm System. Seeds were ground using a sterilized mortar and pestle at room temperature prior to DNA extraction. The mortar and pestle were sterilized with 10% (v/v) bleach solution followed by thorough rinsing with water and ethanol. After removal of the seed coat, the seeds were ground for approximately 3–5 min using a mortar and pestle until a fine, homogeneous powder was obtained. The extent of grinding was visually confirmed by the absence of visible seed fragments and the formation of a uniform powder. Details of all reagents, and instrumentation used in this study can be found in the Supporting Information.

### Choline-based ionic liquids and surfactant syntheses

Detailed information on synthesis of ILs and surfactants and their ^1^H nuclear magnetic resonance spectra can be found in Methods S1 and Figures S16-S20 of the Supporting Information, respectively.

### IL-based DNA extraction

Ground soybean seed powder weighing 100 ± 0.5 mg was placed in a 1.5 mL DNA LoBind tube and mixed with 1 mL aqueous solution of IL in TE buffer and 20 µL of 10 mg/mL RNase A by inverting the tube 10 times. The samples were incubated at 65 °C for 10 min followed by centrifugation for 10 min at 13,000 × g to facilitate phase separation. The supernatant (800 µL) was transferred to a new 2 mL microcentrifuge tube and subjected to spin column purification. For spin column purification, 800 µL of the IL extract was mixed with 900 µL of the binding buffer PC and bound to the spin column by centrifugation at 11,000 × g for 1 min. The spin column was washed with 400 µL of wash buffer PW1 by centrifugation at 11,000 × g for 1 min, 700 µL PW2 by centrifugation at 11,000 × g for 1 min and finally with 200 µL PW2 by centrifugation at 11,000 × g for 2 min. A volume of 30 µL 1X TE buffer was preheated to 65 °C and used for DNA elution after incubation of the spin column at 65 °C for 5 min. Different choline-based ILs and salts were initially screened with 25% (w/v) IL in 1X TE and different weight percentages of ILs in 1X TE buffer were tested during optimization experiments. All extraction experiments were conducted in triplicate.

### Commercial kit-based DNA extraction

Ground soybean seed powder was subjected to CTAB-based lysis using PL1 buffer and SDS-based lysis using PL2 buffer followed by protein precipitation using precipitation buffer PL3 of the NucleoSpin Plant II commercial kit with modifications to the manufacturer’s instructions.

### CTAB-based lysis and purification

Ground soybean seed powder weighing 100 ± 0.5 mg was placed in a 1.5 mL DNA Lobind tube and mixed with 1 mL aqueous solution of PL1 buffer and 20 µL of 10 mg/mL RNase A. The samples were incubated at 65 °C for 10 min. A NucleoSpin^®^ Filter (violet ring) was placed in a new collection tube (2 mL) and the lysate was loaded onto the column. The samples were centrifuged for 2 min at 11,000 x g and 800 µL of the clear flow-through was collected and was transferred to a new 2 mL microcentrifuge tube and subjected to spin column purification, as described in the aforementioned IL-based DNA extraction procedure.

### SDS-based lysis and purification

Ground soybean seed powder weighing 100 ± 0.5 mg was placed in a 1.5 mL DNA LoBind tube and mixed with 1 mL aqueous solution of PL2 buffer and 20 µL of 10 mg/mL RNase A. The samples were incubated at 65 °C for 10 min. To the lysate, 250 µL of buffer PL3 was added and mixed thoroughly and incubated for 5 min on ice to completely precipitate SDS. A NucleoSpin^®^ Filter (violet ring) was placed in a new collection tube (2 mL) and the lysate was loaded onto the column. The samples were centrifuged for 2 min at 11,000 x g and 800 µL of the clear flow-through was collected and transferred to a new 2 mL microcentrifuge tube and subjected to spin column purification, as described in the aforementioned IL-based DNA extraction procedure.

### Quality assessment of DNA

The purity of recovered genomic DNA was evaluated by a NanoDrop spectrophotometer and the A260/280 and A260/230 ratios were assessed. Extracted DNA concentration was measured using a Qubit 4.0 fluorometer (ThermoFisher Scientific, Waltham, MA, USA) with the 1X double-stranded DNA (dsDNA) high sensitivity assay. The DNA samples were diluted to a concentration of 2.0 ± 0.5 ng/µL with 1X TE buffer and 2 µL of the diluted samples were run on the Agilent 5200 Fragment Analyzer^™^ Automated Capillary Electrophoresis system. Data was analyzed by the ProSize version 3.0.1.6 software in NGS analysis mode. The quality of DNA was assessed using DQN which is represented by a numerical score ranging from 1 (poorest) to 10 (highest). DQN is based upon the sample concentration above and below the user defined size threshold (bp) [23]. The threshold was set at 10,000 bp. The HS Large Fragment 50 kb kit (DNF-464-0500) was used for analysis of DNA fragments ranging from 75 to 48,500 bp with the lower and upper markers at 1 bp and 200,000 bp respectively. Sample injection was performed at 5.0 kV for 30 s and the electrophoretic separation was carried out at 5.0 kV for 58 min.

### WGA, SSR and PCR amplification

All details of WGA, PCR and SSR marker amplification conditions as well as the PCR primers and SSR markers used in this study, are provided in the Supporting Information.

### Agarose gel electrophoresis

The PCR products and SSR amplification products of genomic DNA extracted by the IL, CTAB and SDS methods was assessed by agarose gel electrophoresis. A 1% (w/v) agarose gel was prepared with 1X Tris-acetate-EDTA buffer and stained with SYBR^™^ Safe gel stain. To 20 µL of the amplification product, 5 µL of 10% glycerol was mixed and 20 µL of the mixture was loaded on the gel. A 50 bp DNA ladder was used as a reference. Gel electrophoresis was performed on SSR amplicons and PCR products at 70 V for 1 h and 1.5 h, respectively. A Safe Imager 2.0 transilluminator (Invitrogen, Carlsbad, CA, USA) was used to visualize the bands.

### DNA library construction and WGS data analysis

Williams 82 soybean variety was chosen for sequencing due to the availability of the reference genome. DNA extracted from Williams 82 soybean seeds by the IL method was submitted to Novogene Corporation Inc., Sacramento, CA for library preparation and sequencing. Library construction was performed using ABclonal Rapid Plus DNA Library Prep Kit according to manufacturer’s instructions and was sequenced on the NovaSeq X Plus (PE150) platform using a 25B flow cell, aiming for ∼ 30x total genome coverage. Lingering sequencing adapters were removed from Illumina reads using fastp v0.24.0 [32]. Then, the cleaned reads were mapped to a soybean reference genome using BWA-MEM v0.7.17 [33] with default parameters. The reference genome Wm82.a6.v1 [34] which was downloaded from Phytozome 13 [35] was used. Following read mapping, whole genome coverage/depth was compared between unfiltered and filtered alignments with SAMtools v1.16.1 [36]. The final filtered dataset included only primary alignments and those with proper mate pairs, while PCR duplicates and alignments with < 35 mapping quality (MQ) were removed. Bedtools coverage (v2.28.0) [37] was used to calculate read mapping depth across each chromosome in 1 Mb sliding windows (0.9 Mb overlap) for both the filtered and unfiltered datasets, then plotted with karyoploteR v1.16.0 [38].

## Electronic supplementary material

Below is the link to the electronic supplementary material.

Supplementary Material 1

## Data Availability

No datasets were generated or analysed during the current study.
