# Supplementary material for: Ionic liquid-assisted seed genomic DNA extraction for advanced sequencing applications
Source: Plant Methods. 2025 Jul 16;21:97. doi: 10.1186/s13007-025-01417-1 (PMC12265370; doi:10.1186/s13007-025-01417-1)
Supplement: Supplementary file 1 — Supplementary Material 1 [file 13007_2025_1417_MOESM1_ESM.docx]

**Electronic Supporting Information**

**Ionic Liquid-Assisted Seed Genomic DNA Extraction for Advanced Sequencing Applications.**

Shashini De Silva, Philip C. Bentz, Cecilia Cagliero, Morgan R. Gostel,
Gabriel Johnson, and Jared L. Anderson^*^

**Author details**

SD: Department of Chemistry, Iowa State University, Ames, Iowa 50011, USA, email: [shashini@iastate.edu](mailto:shashini@iastate.edu)

PCB: HudsonAlpha Institute for Biotechnology, Huntsville, Alabama, USA, email: [pbentz@hudsonalpha.org](mailto:pbentz@hudsonalpha.org).

CC: Dipartimento di Scienza e Tecnologia del Farmaco, Università di Torino, I-10125, Turin, Italy, email: [cecilia.cagliero@unito.it](mailto:cecilia.cagliero@unito.it)

MRG: Botanical Research Institute of Texas, Fort Worth, Texas 76107-3400, USA, email: [mgostel@brit.org](mailto:mgostel@brit.org)

GJ: Smithsonian Institution, Suitland, Maryland 20746, USA, email: [JohnsonG@si.edu](mailto:JohnsonG@si.edu)

JLA: Department of Chemistry, Iowa State University, Ames, Iowa 50011, USA, email: [andersoj@iastate.edu](mailto:andersoj@iastate.edu)

* Corresponding author:

Jared L. Anderson
Department of Chemistry

Iowa State University
Ames, IA 50011
Tel.: +1 515-294-8356

E-mail address: [andersoj@iastate.edu](mailto:andersoj@iastate.edu)

**Table of Contents**

| Materials | p. S3 |
| --- | --- |
| Method S1 | p. S4 |
| Method S2  Method S2 | p. S5  p. S5 |
| Table S1  Table S2 | p. S7  p. S8 |
| Table S3 | p. S9 |
| Table S4 | p. S10 |
| Table S5 | p. S11 |
| Figure S1 | p. S12 |
| Figure S2 | p. S13 |
| Figure S3 | p. S14 |
| Figure S4 | p. S15 |
| Figure S5 | p. S16 |
| Figure S6 | p. S17 |
| Figure S7 | p. S18 |
| Figure S8 | p. S19 |
| Figure S9 | p. S20 |
| Figure S10 | p. S21 |
| Figure S11 | p. S22 |
| Figure S12 | p. S23 |
| Figure S13 | p. S24 |
| Figure S14 | p. S25 |
| Figure S15 | p. S26 |
| Figure S16 | p. S27 |
| Figure S17 | p. S28 |
| Figure S18 | p. S29 |
| Figure S19 | p. S30 |
| Figure S20 | p. S31 |
| References | p. S32 |

**Materials**

Choline hydroxide solution (46 wt. % in water), dodecanoic acid (≥99%), formic acid (≥95%), 1-bromohexadecane (97%), 2-dimethylaminoethanol (≥99.5%), ethylenediaminetetraacetic acid (EDTA), Amberlite IR120 resin (hydrogen form) and Invitrogen SYBR^TM^ Safe DNA Gel Stain were purchased from Thermo Fischer Scientific (Carlsbad, CA, USA). Acetic acid (glacial, certified ACS), methanol (ACS grade) and agarose (genetic analysis grade), optically clear PCR caps and tube strips were acquired from Thermo Fisher Scientific (Waltham, MA, USA). Tris(hydroxymethyl)aminomethane HCl (>99.0%) was purchased from RPI Corp. (Mount Prospect, IL, U.S.A.) Lambda DNA (N30112) was purchased from New England BioLabs (Ipswich, MA, USA). All qPCR primers and SSR markers, shown in Table S5, were purchased from Integrated DNA Technologies (Coralville, IA, USA). Phosphoric acid, lyophilized RNase A (Macherey–Nagel, Düren, Germany), and a NucleoSpin Plant II commercial kit (Macherey–Nagel, Düren, Germany) were purchased from Fisher Scientific. A 50 bp DNA ladder was purchased from Gold Biotechnology (St Louis, MO, USA). A Qiagen REPLI-g ultrafast mini kit (containing buffers D1 and N1, mastermix and polymerase) was received as a gift from Qiagen (Valencia, CA, USA). Agarose gel electrophoresis was carried out using a Bethesda Research Laboratories H4 Horizontal Gel Electrophoresis system (Life Technologies) and a dual output power supply (Neo/Sci, Rochester, NY, USA). A Milli-Q water purification system (Bedford, MA, USA) was used to supply 18.2 MΩ•cm deionized water for the preparation of aqueous solutions. An Accumet AB-150 pH meter from Fisher Scientific was used for all pH measurements. An agate mortar (100 mm O.D. × 82 mm I.D. × 25 mm depth) with a pestle acquired from MSE supplies (Tucson, AZ, USA) was used for sample grinding. Eppendorf DNA LoBind 1.5 mL tubes (Hamburg, Germany) were employed for all DNA extraction experiments and storage. For DNA purification from IL extracts, binding buffer (PC) and wash buffers (PW1 and PW2) from Macherey–Nagel, Düren, Germany were used.

**Methods S1: Choline-based ionic liquids and surfactant syntheses**

Synthesis of choline-based ionic liquids and salts were performed based on previously reported procedures.[1] The exact concentration of 46 wt% choline hydroxide solution was determined by a titration with standardized hydrochloric acid prior to use. Choline formate, choline acetate, and choline dodecanoate were prepared by the dropwise addition of aqueous choline hydroxide to the corresponding acid (1:1) in water or methanol and stirred at ambient temperature overnight. Water or solvent was then removed under reduced pressure, first using rotary evaporation (e.g., 50 °C), and then dried under vacuum in a vacuum oven for 3 days.

Choline dodecyl sulfate was prepared based on a previously reported procedure, with slight modification.[2] Ion exchange of sodium dodecyl sulfate was performed using Amberlite IR120 resin in the hydrogen ion form. First, the column was charged with 1 M HCl and washed with a large amount of water until a near-neutral pH was achieved. An aqueous solution of choline chloride (1 M, corresponding to four times the maximum cation exchange capacity) was passed over the resin until the pH value of the effluent was decreased to approximately 4–5. After the addition of choline chloride solution, the column was rinsed with water and the effluent was tested with silver nitrate. Sodium dodecyl sulfate (SDS) was passed through the column as a 0.1 M solution that corresponds to lower than 1/3 of the minimum resin capacity. After discarding forerunnings of the column, the effluent was collected and solid surfactants were obtained by lyophilization followed by drying of the white powders in a desiccator.

Synthesis of the modified choline bromide salt was based a previously reported procedure.[3] The reagents 1-bromohexadecane and 2- (dimethylamino)ethanol were mixed in hexane and refluxed at 80 °C for 8−10 h. The white solid precipitate was then filtered and washed with hexane. The product was dried under reduced pressure and stored in a vacuum oven.

**Method S2: Whole genome amplification (WGA) conditions**

WGA was performed using the Qiagen REPLI-g ultrafast mini kit according to the manufacturer’s instructions. To 1 μL of purified genomic DNA having a concentration of 2 ng/μL, 1 μL of buffer D1 (prepared using 5 μL of reconstituted buffer DLB of the Qiagen REPLI-g ultrafast mini kit and 35 μL water) was added and mixed. The samples were incubated at room temperature for 3 min and 2 μL of buffer N1 (prepared using 8 μL of stop solution of the Qiagen REPLI-g ultrafast mini kit and 72 μL water) was added and mixed. To 4 μL of the denatured DNA, 16 μL of the master mix (prepared using 15 μL of REPLI-g ultrafast reaction buffer and 1 μL REPLI-g ultrafast DNA polymerase) was added and incubated at 30 °C for 1.5 h. To inactivate the DNA polymerase, the sample was heated for 3 min at 65 °C. WGA products were quantified using the Qubit 1X dsDNA high sensitivity assay and fragment sizes were analyzed by an Agilent 5200 Fragment Analyzer^TM^ Automated Capillary Electrophoresis system.

**Method S3: SSR and PCR amplification conditions**

The SSR markers Satt181, Satt 157s, Satt 357s, and Satt373 were amplified using DNA extracted from soybean seeds using the IL, CTAB, and SDS methods with specific primers (Table S5). All reactions were performed using a Bio-Rad CFX96 Touch Real-time PCR thermocycler (Hercules, CA, USA) with a total volume of 20 μL. Each reaction containing 1 μL of the DNA required 10.0 μL of SsoAdvanced Universal SYBR Green Supermix, 8.6 μL of deionized water and 0.4 μL of 10 μM of each primer. The thermocycling conditions involved an initial denaturation step of 2 min at 95 °C and 40 cycles comprised of a 30 s denaturation step at 95 °C, a 30 s annealing step at 60 °C and a 30 s extension step at 72 °C followed by an optical detection step. Final extension was carried out at 72 °C for 3 min. Melt curve analysis was carried out after amplification and began at 65 °C for 5 s while increasing to 95 °C in 0.5 °C increments.

Real-time amplification of nuclear and plastid DNA regions was performed at 95 °C for 10 min, 40 cycles of 95 °C for 15 s, and 60 °C for 45 s followed by an optical detection step. Melt curve analysis was carried out after amplification and began at 65 °C for 5 s while increasing to 95 °C in 0.5 °C increments. Each reaction containing 1 μL of the DNA required 10.0 μL of SsoAdvanced Universal SYBR Green Supermix, 8.6 μL of deionized water, and 0.4 μL of 10 μM of each primer**.**

**Table S1.** Summary of statistical comparisons between neat choline formate (CF) or neat choline acetate (CA) and their respective aqueous mixtures (10%, 25%, 50%, and 75% w/v) for DNA yield and abundant DNA fragment size. Statistical significance was determined using the Student’s t-test (N = 3). n.s. (no significance); *p < 0.05, **p < 0.01, ***p < 0.001, ****p < 0.0001

| **Comparison** | **DNA yield** | **Abundant fragment size** |
| --- | --- | --- |
| Neat CF vs. 10% CF | ******** | ****** |
| Neat CF vs. 25% CF | ****** | ****** |
| Neat CF vs. 50% CF | ******** | ***** |
| Neat CF vs. 75% CF | ****** | ***** |
| Neat CA vs. 10% CF | ***** | ****** |
| Neat CA vs. 25% CF | ***** | ***** |
| Neat CA vs. 50% CF | ***** | ***** |
| Neat CA vs. 75% CF | ***** | ****** |

**Table S2.** Comparison of DNA yield and quality metrics before and after WGA using the IL, CTAB, and SDS extraction methods.

|  | **Before WGA** | | | **After WGA** | | | | **Significance of percentage increase in yield (vs. IL method)** |
| --- | --- | --- | --- | --- | --- | --- | --- | --- |
| **Extraction method** | **Concentration (ng/μL)** | **DQN** | **Abundant fragment size (bp)** | **Concentration (ng/μL)** | **DQN** | **Abundant fragment size (bp)** | **Percentage increase in yield** |  |
| IL method | 2.46 ± 0.18 | 6.6 ± 0.2 | 23880 ± 1068 | 23.0 ± 7.80 | 0.6 ± 0.0 | 3151 ± 236 | 928 ± 274 | - |
| CTAB method | 2.17 ± 0.18 | 7.0 ± 0.7 | 16985 ± 4823 | 117 ± 18.6 | 2.6 ± 0.1 | 6129 ± 644 | 5456 ± 1251 | ** |
| SDS method | 1.86 ± 0.08 | 6.7 ± 0.3 | 14719 ± 246 | 22.0 ± 9.90 | 1.1 ± 0.1 | 3324 ± 161 | 1196 ± 581 | n.s. |

Statistical significance determined using the Student’s t-test comparing each method to the IL method: p <0.01 (**), n.s. = not significant.

**Table S3:** Coverage, depth, and quality metrics resulting from read-mapping of Illumina sequencing data for both filtered and unfiltered reads for each chromosome.

| **Chromosome** | **Unfiltered coverage (%)** | **Filtered coverage (%)** | **Unfiltered depth** | **Filtered depth** | **Mean base quality** |
| --- | --- | --- | --- | --- | --- |
| Gm01 | 99.8 | 95.7 | 46.8 | 40.9 | 39.6 |
| Gm02 | 99.8 | 93.7 | 42.2 | 36.5 | 39.6 |
| Gm03 | 99.8 | 94.4 | 40.0 | 35.0 | 39.6 |
| Gm04 | 99.8 | 93.3 | 42.7 | 36.9 | 39.6 |
| Gm05 | 99.8 | 95.4 | 44.0 | 38.0 | 39.6 |
| Gm06 | 99.8 | 94.6 | 40.8 | 35.4 | 39.6 |
| Gm07 | 99.8 | 95.1 | 42.7 | 37.5 | 39.6 |
| Gm08 | 99.8 | 95.2 | 39.9 | 35.6 | 39.6 |
| Gm09 | 99.8 | 95.3 | 45.3 | 38.8 | 39.6 |
| Gm10 | 99.8 | 94.5 | 40.4 | 34.9 | 39.6 |
| Gm11 | 99.8 | 94.9 | 38.9 | 34.0 | 39.6 |
| Gm12 | 99.8 | 94.7 | 57.2 | 40.9 | 39.6 |
| Gm13 | 99.8 | 96.8 | 42.9 | 35.6 | 39.6 |
| Gm14 | 99.8 | 95.8 | 45.7 | 40.1 | 39.6 |
| Gm15 | 99.8 | 92.3 | 50.0 | 41.4 | 39.6 |
| Gm16 | 99.7 | 94.4 | 43.6 | 38.3 | 39.6 |
| Gm17 | 99.8 | 95.4 | 45.7 | 40.9 | 39.6 |
| Gm18 | 99.8 | 95.3 | 50.5 | 43.8 | 39.6 |
| Gm19 | 99.8 | 93.8 | 41.0 | 35.7 | 39.6 |
| Gm20 | 99.8 | 95.5 | 41.8 | 37.3 | 39.6 |

**Table S4**. Summary of yield and NanoDrop UV absorbance ratios for DNA extracted from maize seeds by the three different extraction methods examined in this study.

| **Sample** | **Extraction method** | **Yield**  **(ng/μL)** | **Absorbance 260/280 nm** | **Absorbance 260/230 nm** |
| --- | --- | --- | --- | --- |
| Maize seeds | IL method | 16.1 ± 2.86 | 2.04 ± 0.06 | 1.70 ± 0.22 |
|  | CTAB method | 1.57 ± 0.07 | 0.53 ± 0.58 | 2.79 ± 6.23 |
|  | SDS method | 19.9 ± 0.46 | 2.08 ± 0.02 | 1.66 ± 0.25 |
|  |  |  |  |  |

**Table S5:** Sequences of the primers used in this study.

| **Name** | **Sequence** | **Reference** |
| --- | --- | --- |
| *rbcLa* Forward  *rbcLa* Reverse | ATGTCACCACAAACAGAGACTAAAGC  GTAAAATCAAGTCCACCRCG | [4] |
| Nuclear DNA Forward  Nuclear DNA Reverse | GAGAAGAGTATCCGGATAGG  GAGCTTGAGTGTTCGGAAAC | [5] |
| Plastid DNA Forward  Plastid DNA Reverse | CTTCTACAACCCCTGATATTCAAAG  ACATACATAATAAGATGTGAATGATAC | [5] |
| Satt357s  Satt357as | CCTGAGCAATTCATACTCC  TAACCGATCCGATCCTTGACA | [6] |
| Satt157s  Satt157as | GGGCTCACTCTCGATAGTAGGTATAAAG  GGGATACCAAAAGGAATAATTGTCTT | [6] |
| Satt181F  Satt181R | TGGCTAGCAGATTGACA  GGAGCATAGCTGTTAGGA | [5] |
| Satt373F  Satt373R | TCCGCGAGATAAATTCGTAAAAT  GGCCAGATACCCAAGTTGTACTTGT | [5] |

**
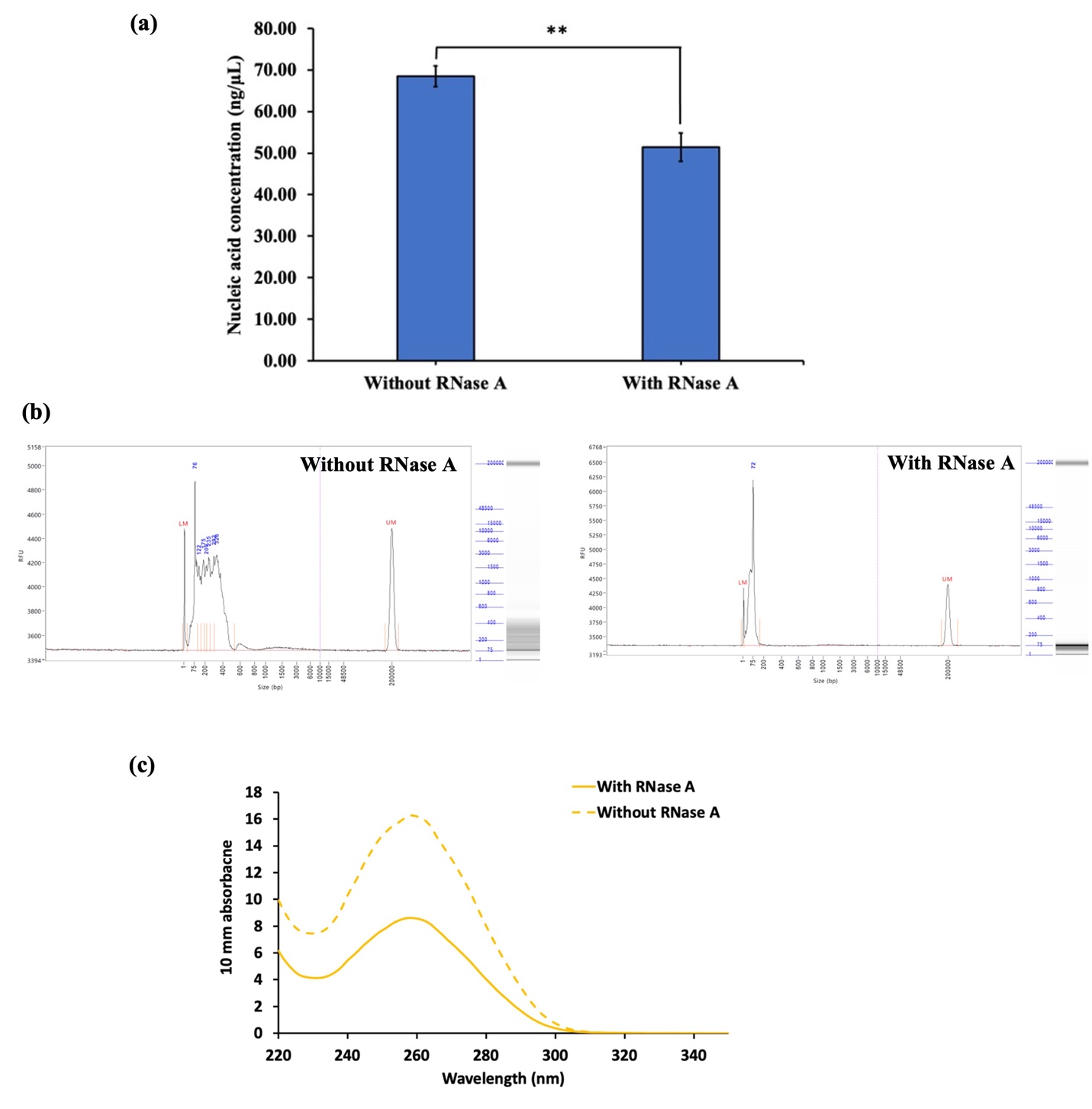
**

**Figure S1.** Effect of RNase A with neat choline formate. (a) Nucleic acid yield (b) Representative electropherogram plots from Fragment Analyzer (c) UV absorbance spectra for recovered soybean DNA after extraction with neat choline formate IL in the absence and presence of RNase A. Statistical significance was determined using the Student’s t-test (N = 3). **p < 0.01. Error bars represent the standard deviation.

**
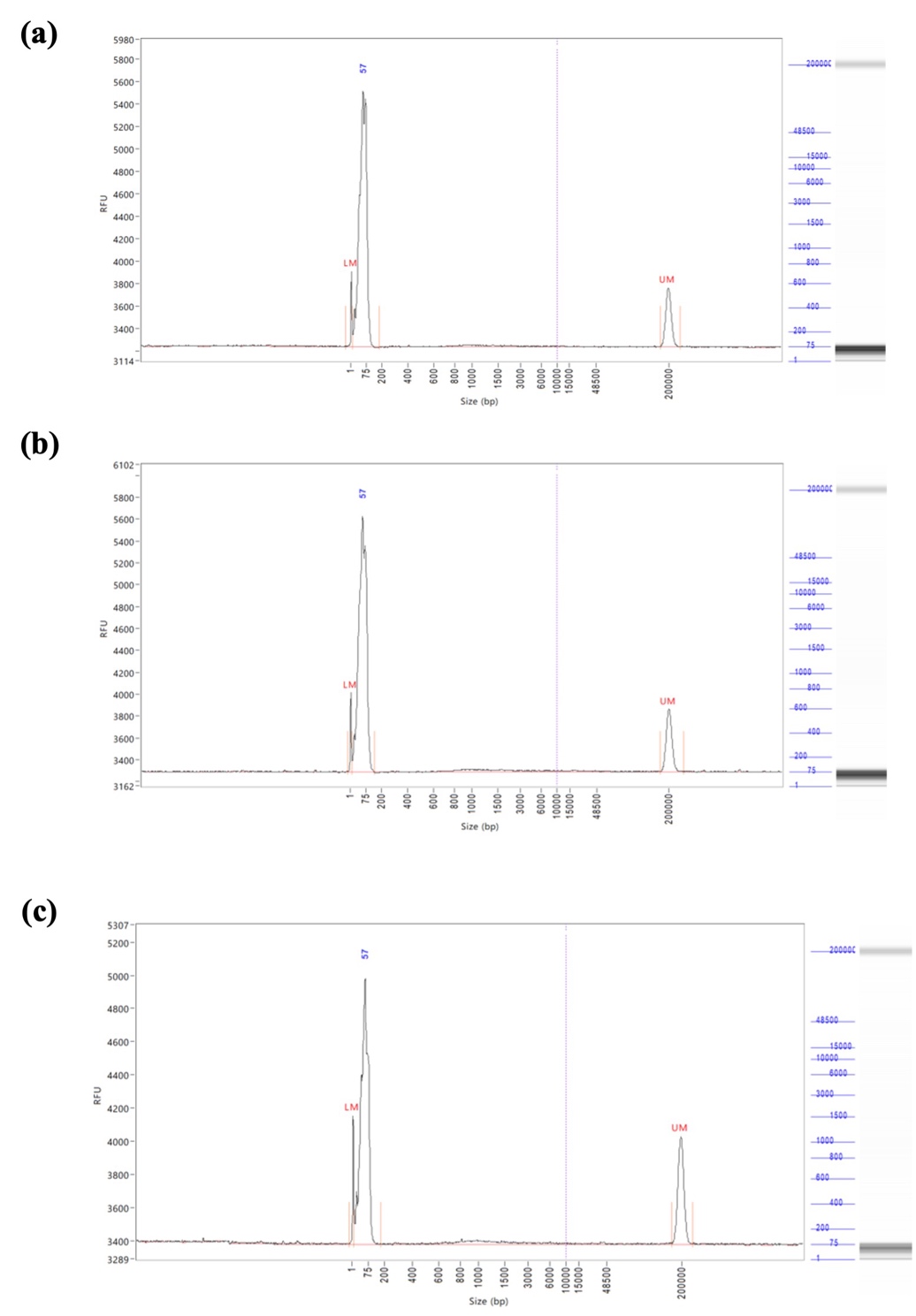
**

**Figure S2.** Fragment analysis of recovered soybean DNA after extraction with neat ILs. Representative electropherograms for: (a) first eluted fraction, (b) second eluted fraction, and (c) eluted fraction after reapplication of the IL solution flowthrough from the initial binding step followed by washing steps.


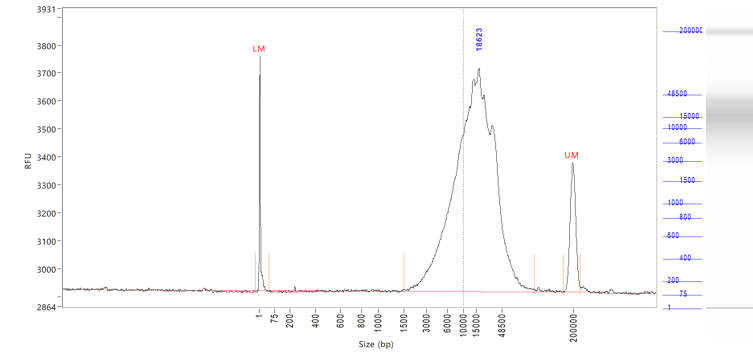


**Figure S3.** Fragment analysis of lambda DNA spiked in neat choline formate IL followed by spin column purification.

.


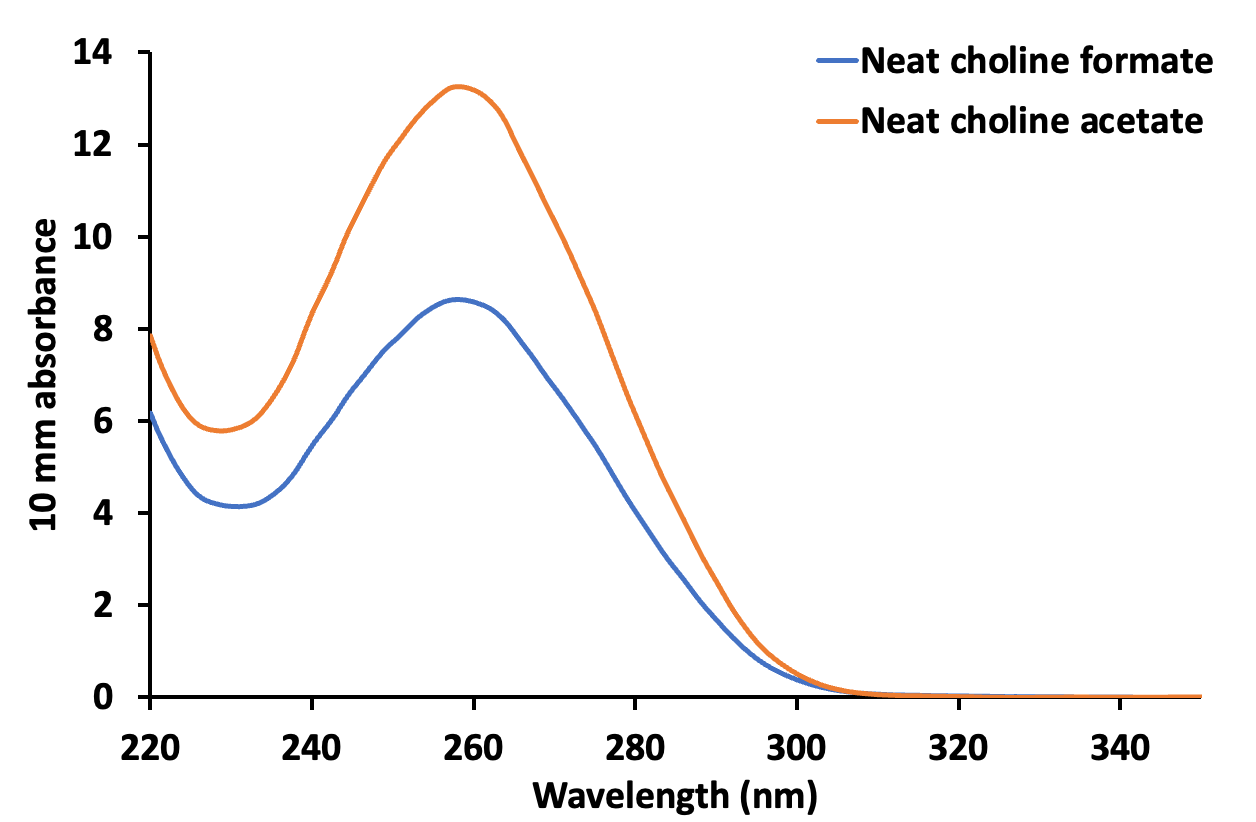


**Figure S4.** UV absorbance spectra for recovered soybean DNA after extraction with neat choline formate IL and choline acetate ILs in the presence of RNase A followed by spin column purification.


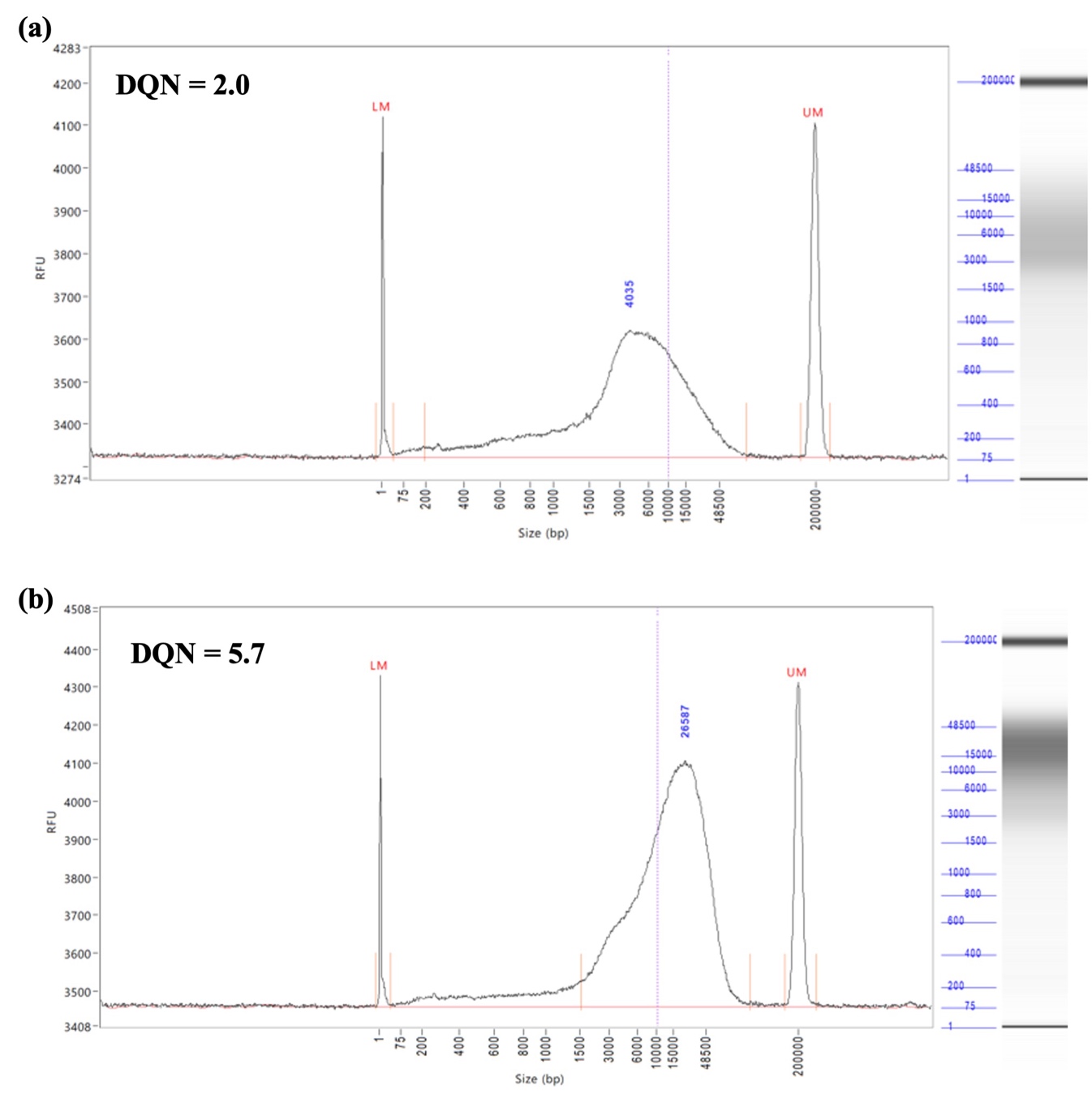


**Figure S5.** Effect of proteinase K treatment. Representative electropherograms from the Fragment Analyzer for recovered soybean DNA after extraction with (a) neat 1X TE buffer (b) 25 % (w/v) choline formate in 1X TE buffer followed by the treatment with proteinase K and spin column purification.


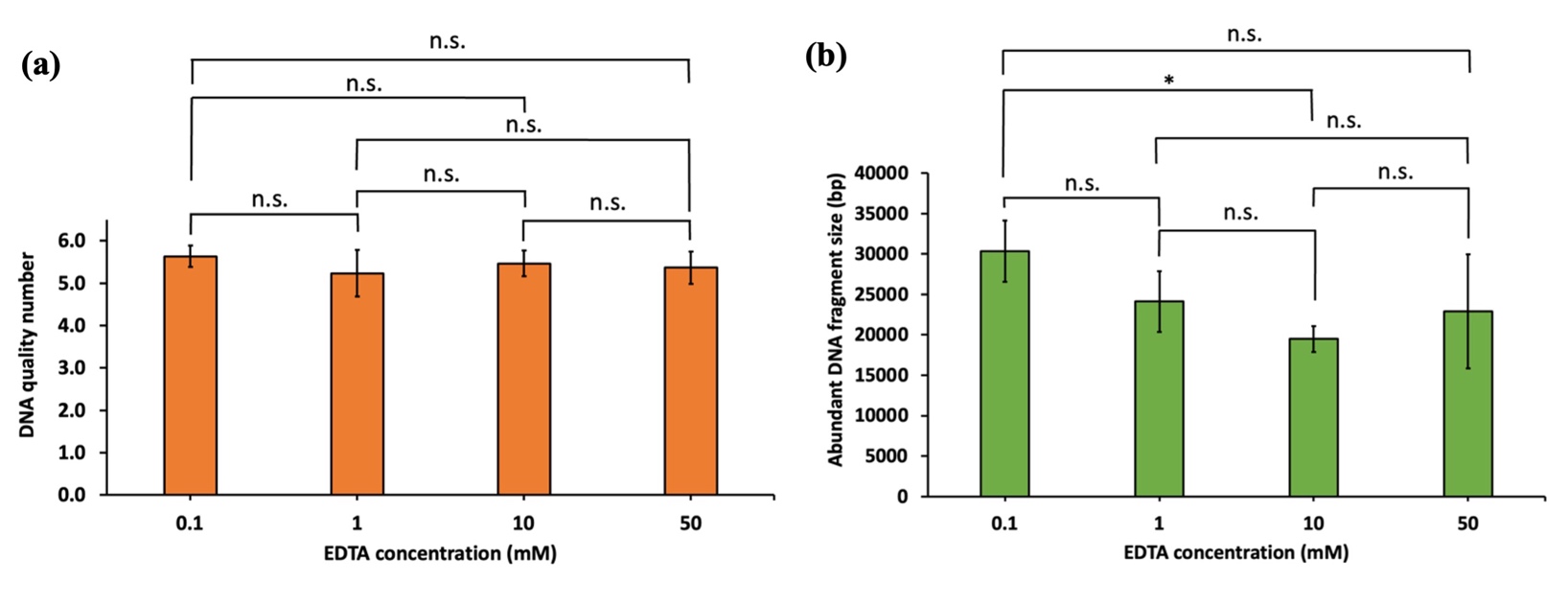


**Figure S6.** Effect of EDTA concentration in the buffer containing 25% (w/v) choline formate and 10 mM Tris-HCl on (a) DNA integrity (orange bars) and (b) abundant fragment sizes (green bars). Ground soybean powder of 100 mg weight was mixed with 1 mL of IL-buffer solution and 20 μL of 10 mg/mL RNase A, incubated at 65 °C for 10 min, centrifuged and the supernatant subjected to spin column purification. Statistical significance was determined using the Student’s t-test (N = 3). n.s. (no significance); *p < 0.05, **p < 0.01, ***p < 0.001, ****p < 0.0001. Error bars represent the standard deviation.


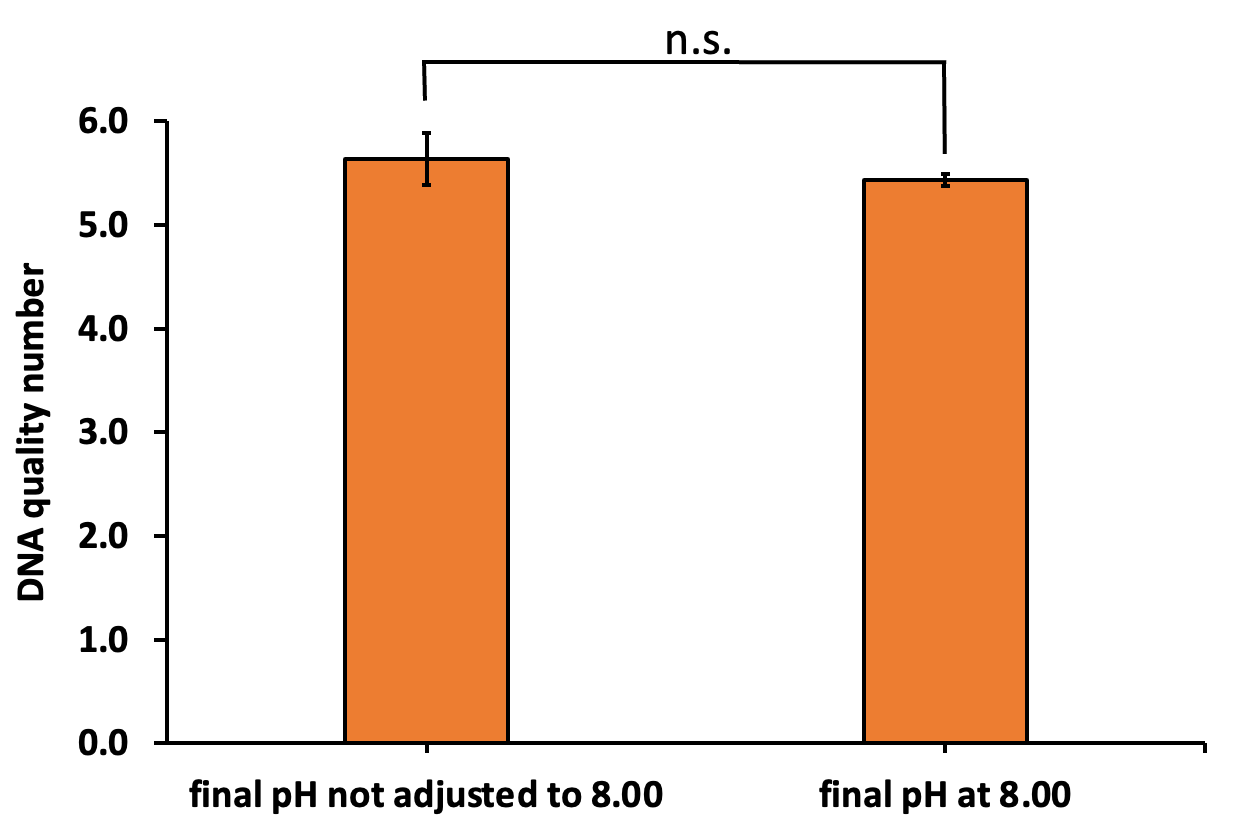


**Figure S7.** Effect of pH adjustment of the buffer. DNA integrity in terms of DNA quality number for recovered soybean DNA after extraction with (a) 25 % (w/v) choline formate dissolved in 1X TE buffer of pH 8.0 (final pH unadjusted) (b) 25 % (w/v) choline formate in 1X TE buffer (final pH adjusted to 8.0) followed by spin column purification. Statistical significance was determined using the Student’s t-test (N = 3). n.s. (no significance); Error bars represent the standard deviation.


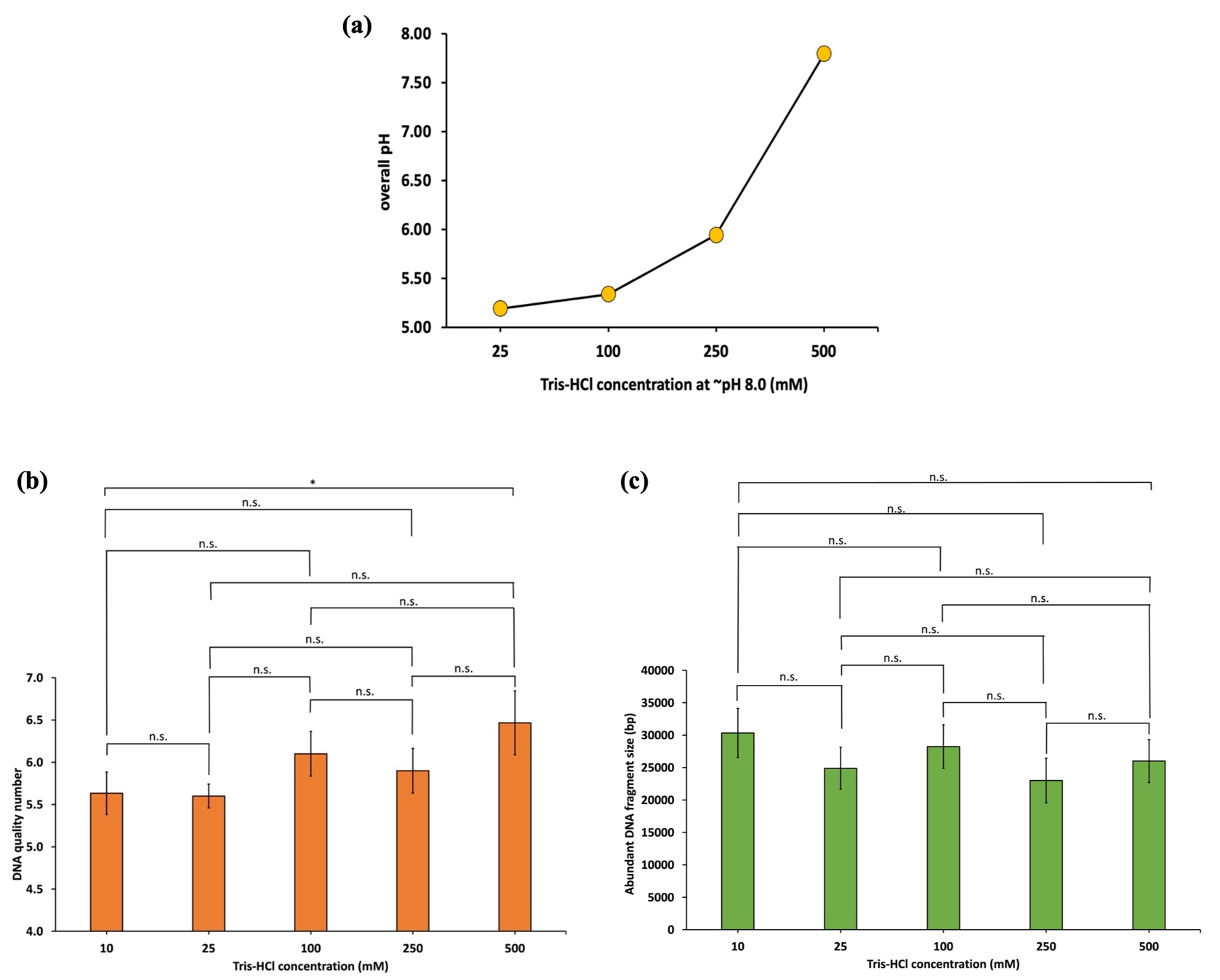


**Figure S8.** Effect of Tris-HCl concentration at pH 8.0 on (a) the pH adjustment of aqueous solution containing 25% (w/v) choline formate and 0.1 mM EDTA. (b) Effect of Tris-HCl buffer concentration at pH 8.0 in the buffer containing 25% (w/v) choline formate and 10 mM Tris-HCl on DNA integrity (orange bars) and (c) abundant fragment sizes (green bars). Ground soybean powder of 100 mg weight was mixed with 1 mL of IL-buffer solution and 20 μL of 10 mg/mL RNase A, incubated at 65 °C for 10 min, centrifuged and the supernatant subjected to spin column purification. Statistical significance for extraction experiments was determined using the Student’s t-test (N = 3). n.s. (no significance); *p < 0.05. Error bars represent the standard deviation.


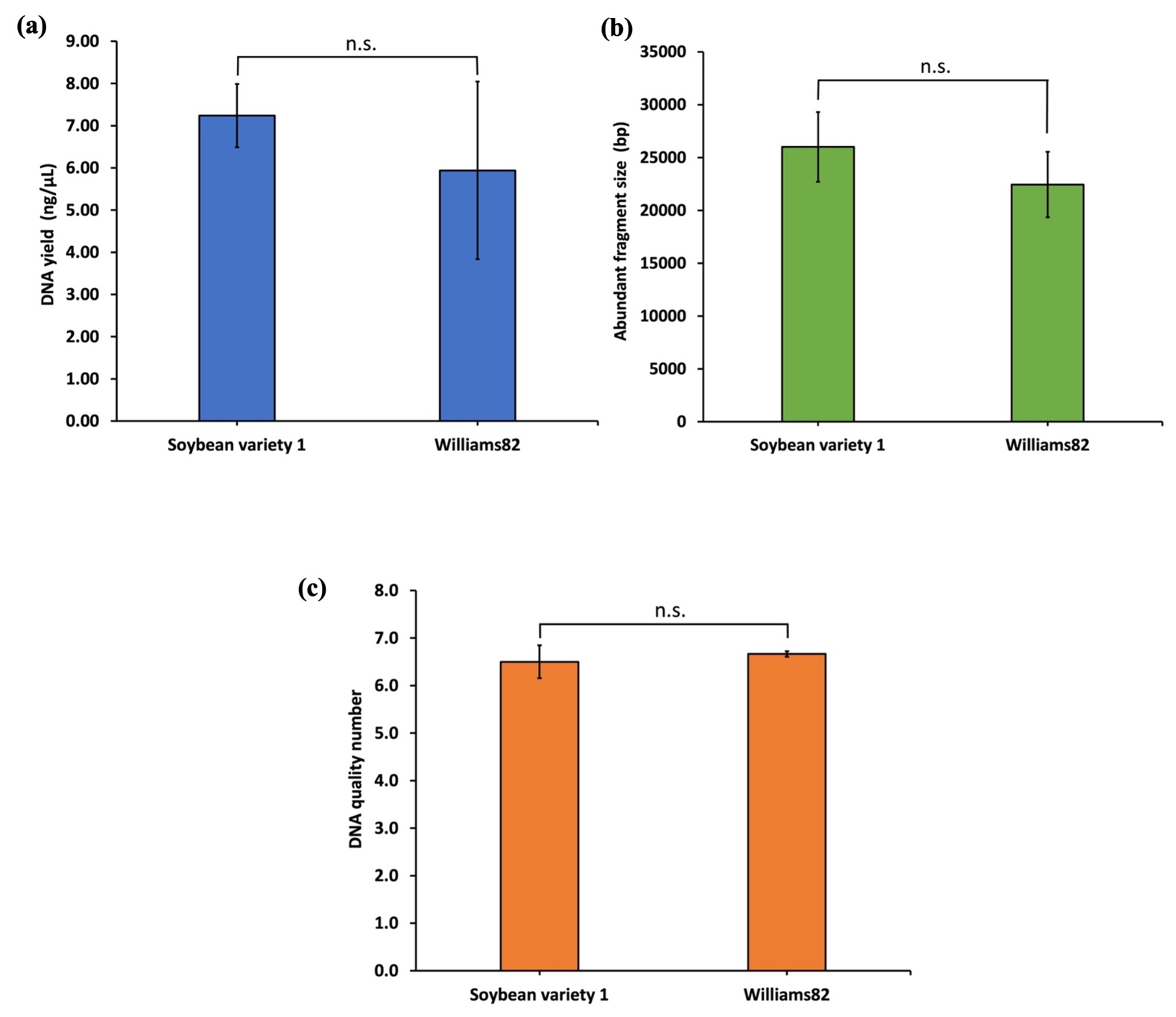


**Figure S9.** IL-based extraction method for other soybean varieties. (a) DNA yield, (b) abundant fragment size, and (c) DNA integrity for different soybean varieties subjected to IL extraction. Statistical significance for extraction experiments was determined using the Student’s t-test (N = 3). n.s. (no significance). Error bars represent the standard deviation.


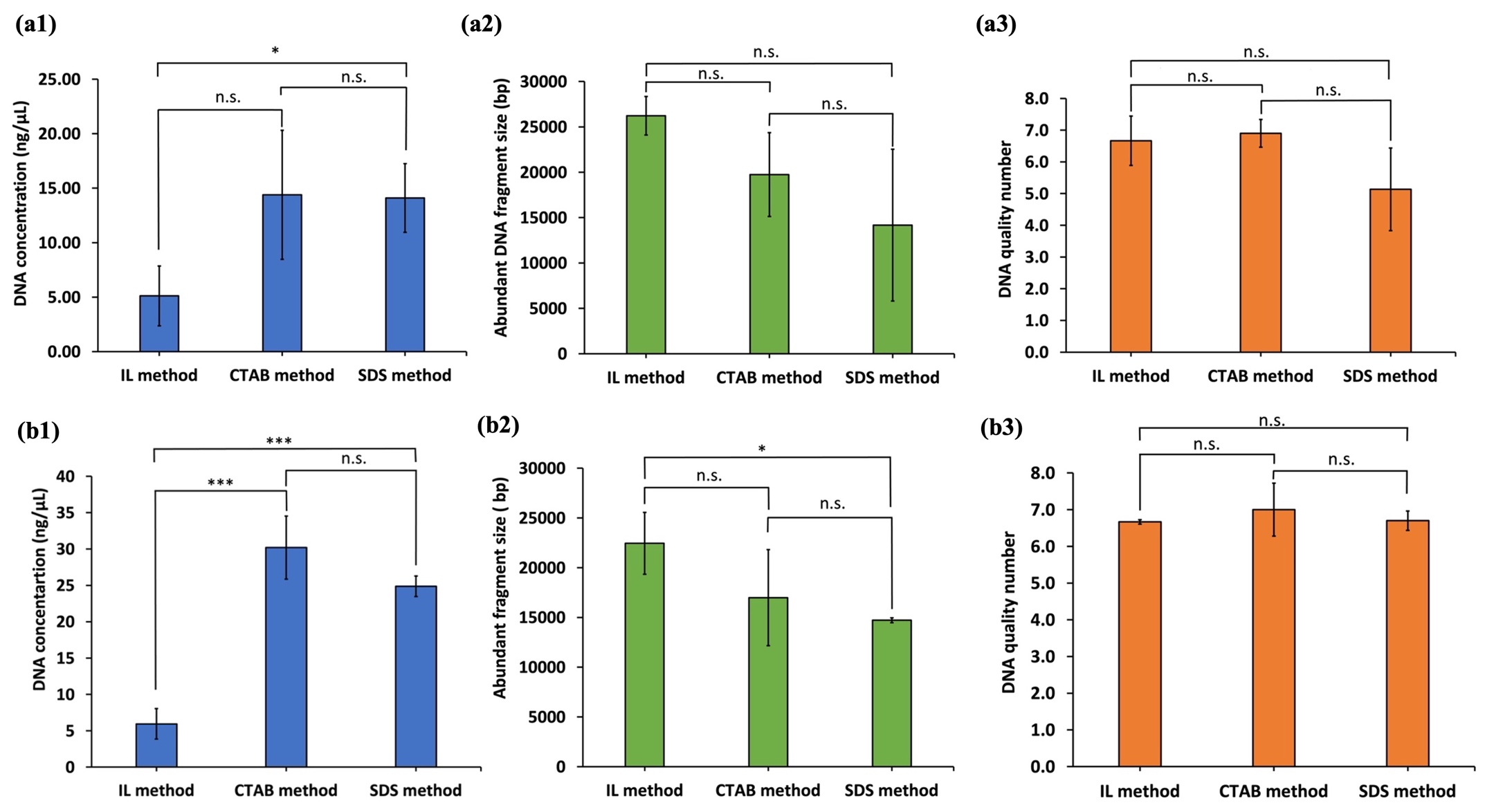


**Figure S10.** Comparison of IL-based DNA extraction method with the CTAB and SDS method for (a) soybean variety 1 and (b) Williams82 in terms of DNA yield (blue bars), abundant fragment sizes (green bars) and DNA integrity (orange bars). Statistical significance was determined using the Student’s t-test (N = 3). n.s. (no significance); *p < 0.05, **p < 0.01, ***p < 0.001, ****p < 0.0001. Error bars represent the standard deviation.


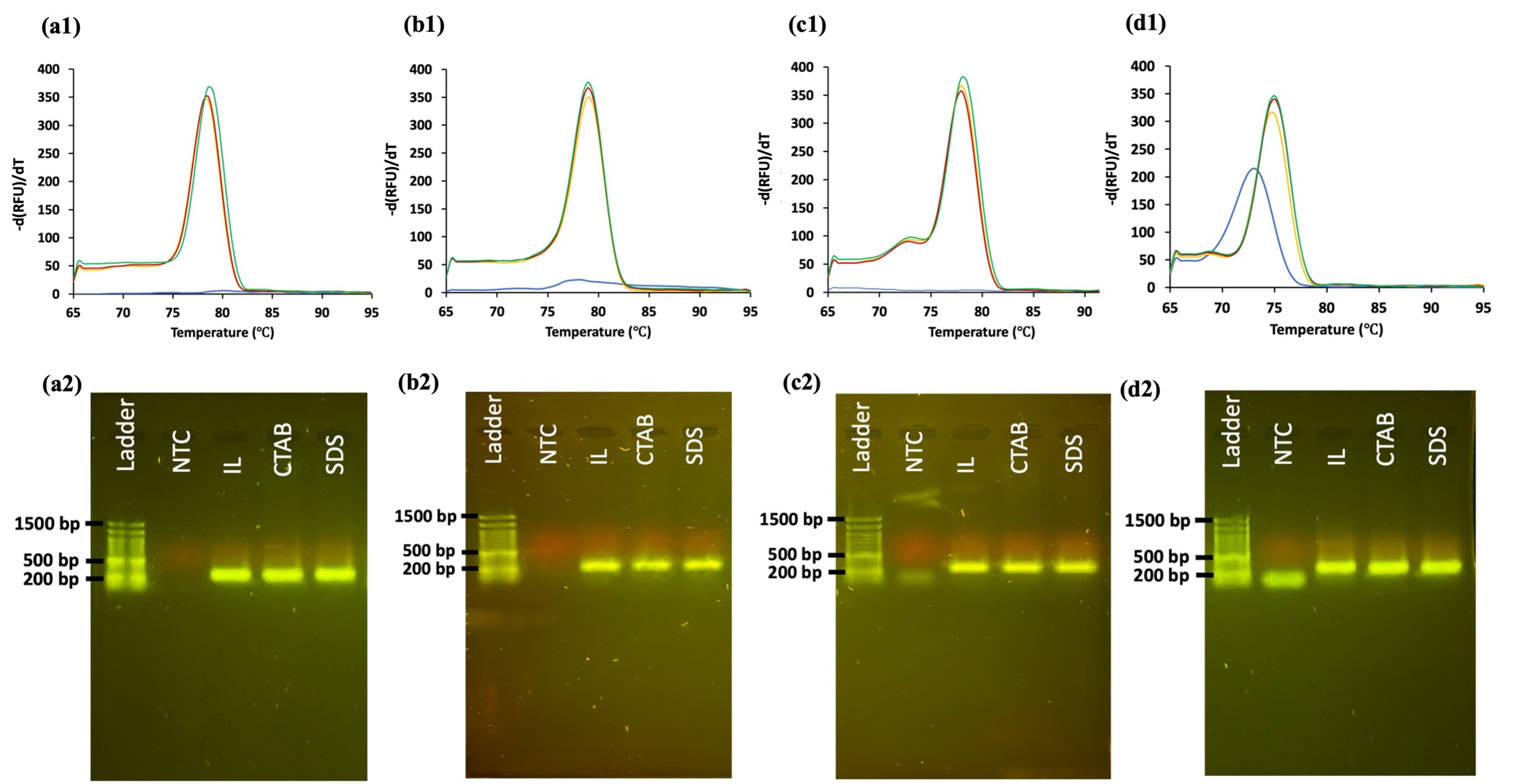


**Figure S11.** Melt curve plots and agarose gel electrophoresis images for SSR marker amplification products. Melt curve plots (top (a1) -(d1)) and agarose gel electrophoresis images (bottom (a2)-(d2)) for Williams82 soybean DNA extracted by IL (yellow), CTAB (red) and SDS (green) methods and amplified by real-time PCR using the following soybean SSR markers, (a) Satt181(b) Satt357s (c) Satt373 (d) Satt157s; Ladder: 50 bp molecular size marker, NTC is in blue.


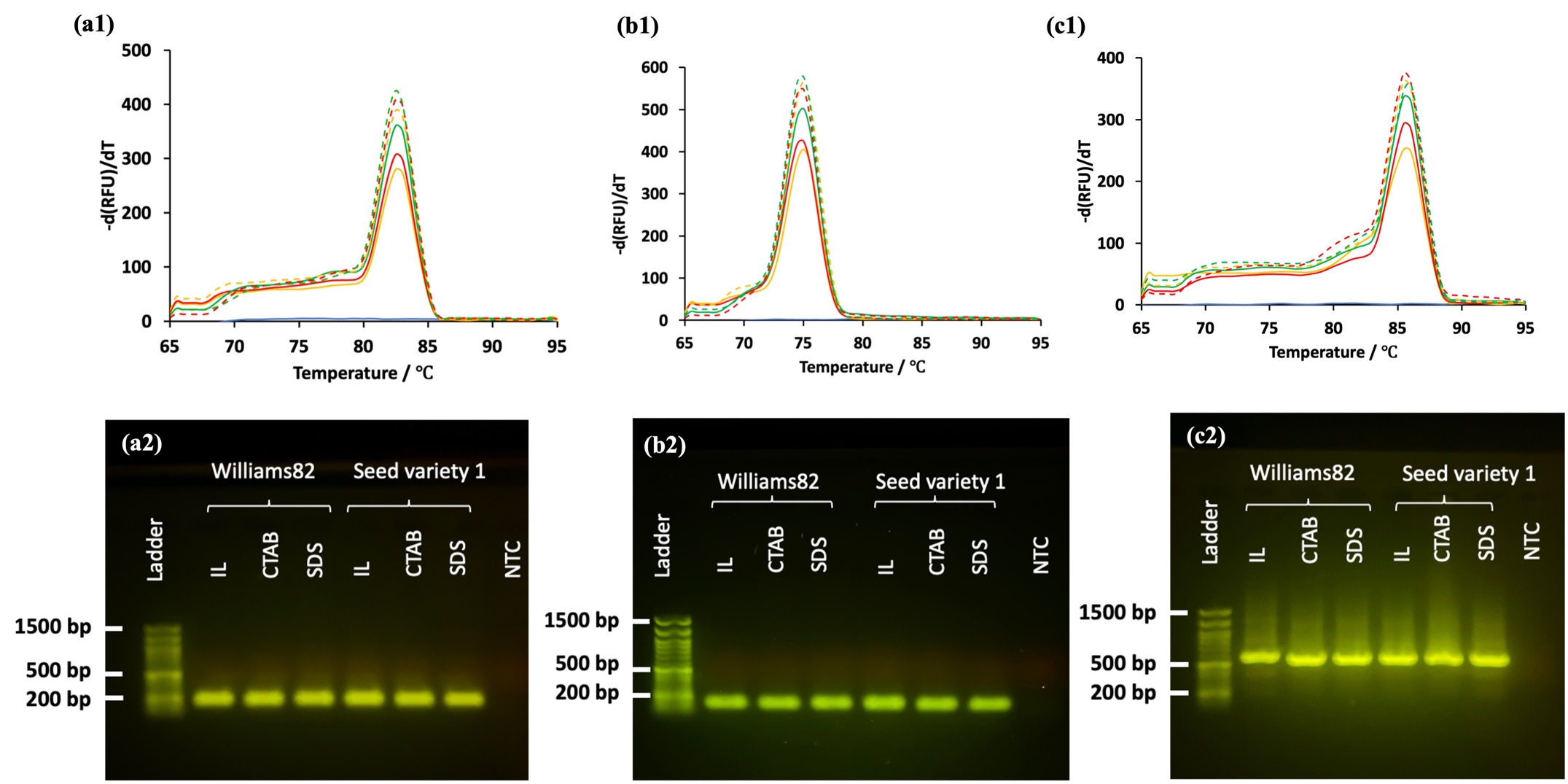


**Figure S12.** Melt curve plots and agarose gel electrophoresis images for PCR amplicons**.** Melt curve plots (top (a1)-(c1)) and agarose gel electrophoresis images (bottom (a2)-(c2)) for PCR amplicons derived from the real-time amplification of DNA extracted using the IL (yellow), CTAB (red), and SDS (green) methods for Williams 82 (solid lines) and soybean variety 1 (dashed lines) using specific primers targeting (a) nuclear DNA, (b) chloroplast DNA, and (c) the *rbcL* gene. Ladder: 50 bp molecular size marker.

**Figure S13.** Illumina read (filtered) mapping depth across all pseudo-chromosome assemblies from the soybean reference Wm82.a6.v1 [7]. Depth was summarized in 1 Mb sliding windows (0.9 Mb overlap) and plotted with karyoploteR v1.16.0 [8]. A maximum cutoff of 70x depth was applied for illustration purposes.


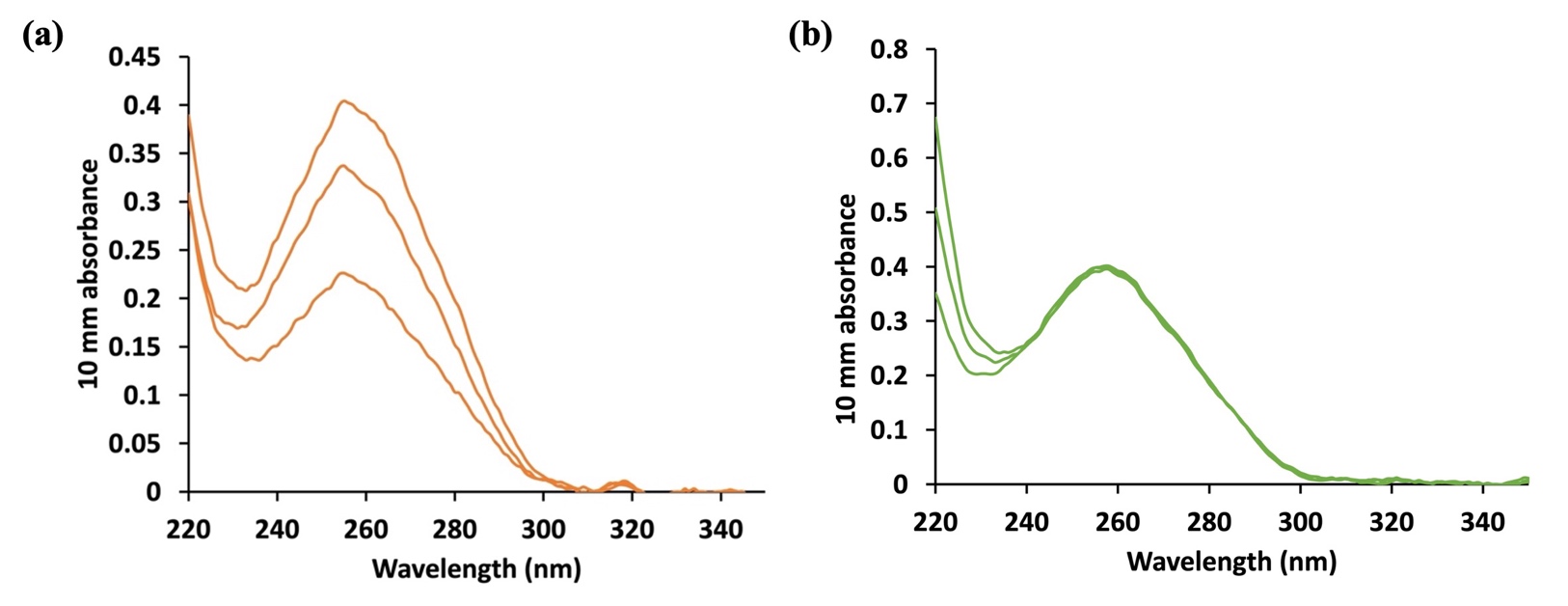


**Figure S14.** NanoDrop UV absorption spectra for DNA extracted from maize seeds by **(a)** IL and **(b)** SDS methods.


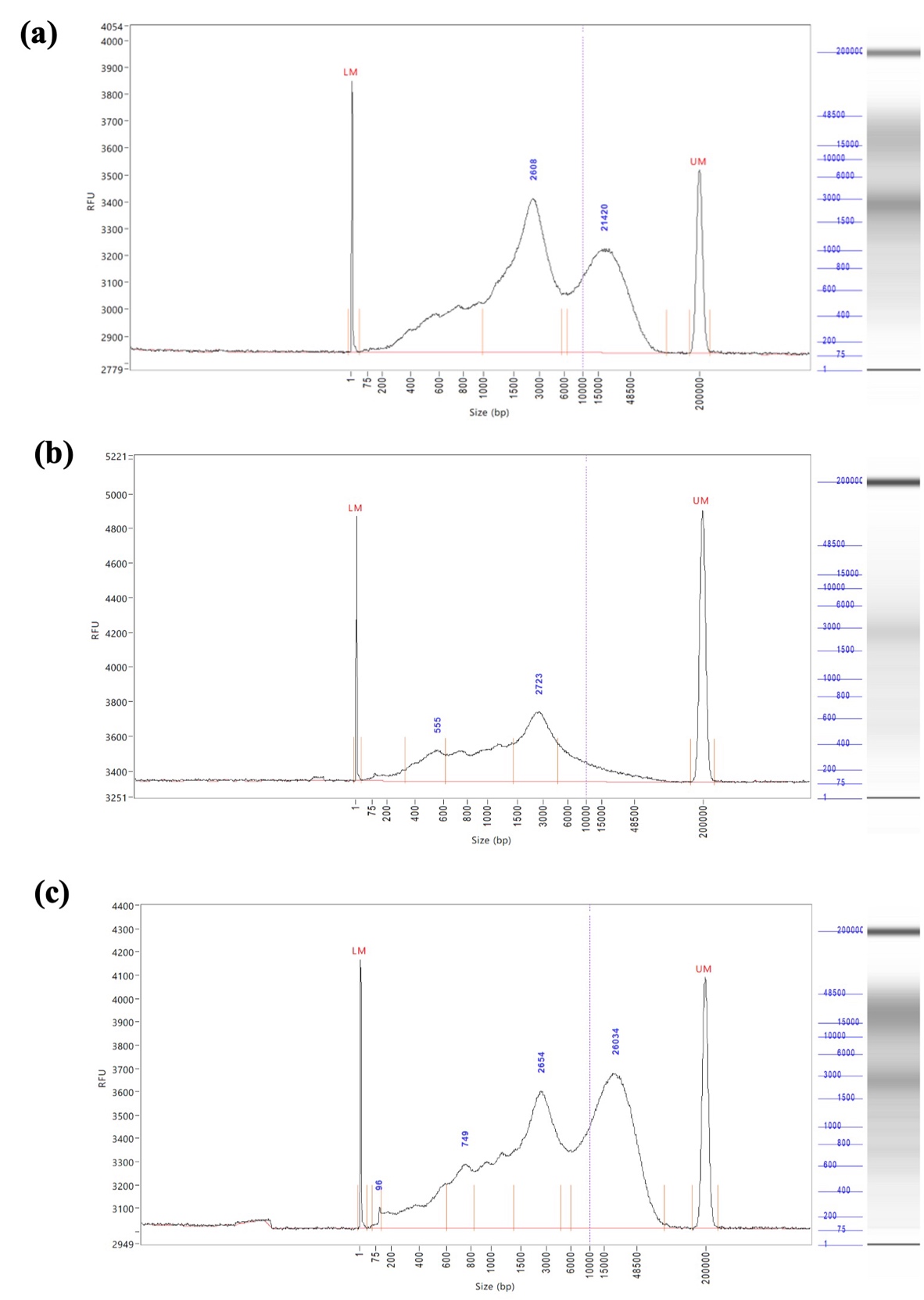


**Figure S15.** Representative electropherograms for DNA extracted from maize seeds by (a) IL method, (b) CTAB method, and (c) SDS method.


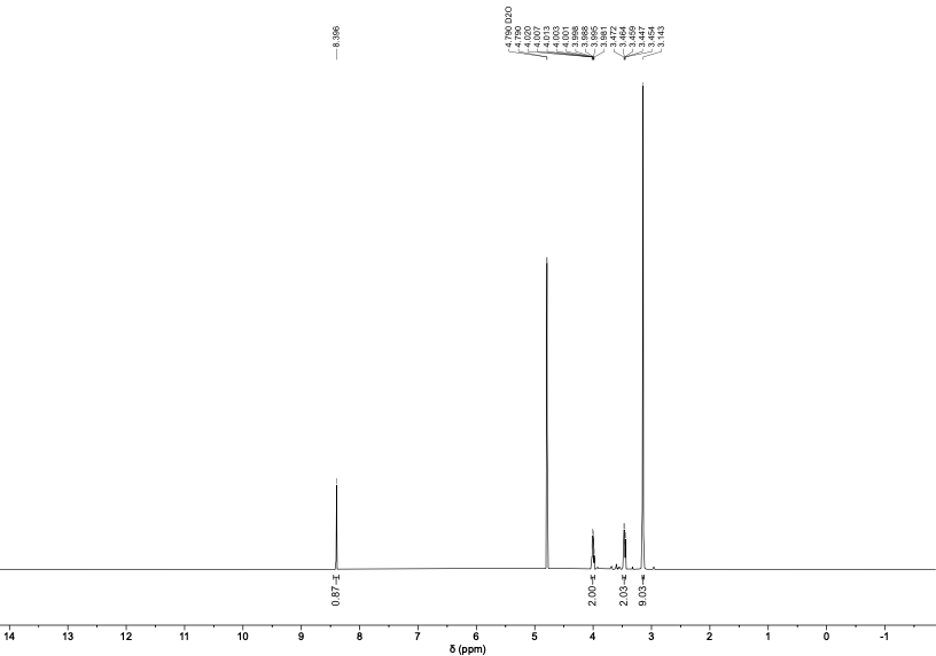


**Figure S16.** 1H NMR of choline formate

1H NMR (400 MHz, D_2_O) δ 8.40 (s, 1H), 4.04 – 3.98 (m, 2H), 3.49 – 3.44 (m, 2H), 3.14 (s, 9H).


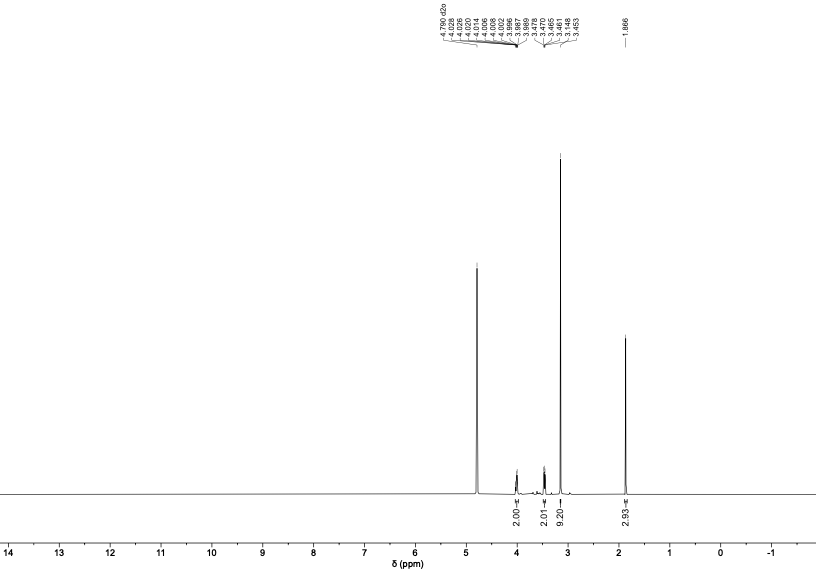


**Figure S17.** 1H NMR of choline acetate

1H NMR (400 MHz, D_2_O) δ 4.04 – 3.98 (m, 2H), 3.49 – 3.44 (m, 2H), 3.15 (s, 9H), 1.87 (s, 3H).


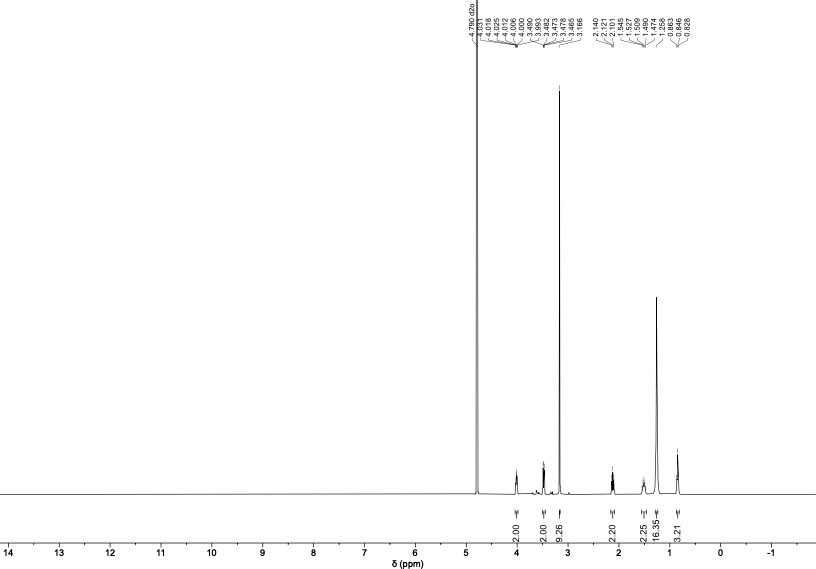


**Figure S18.** 1H NMR of choline dodecanoate

^1^H NMR (400 MHz, D_2_O) δ 4.04 – 3.98 (m, 2H), 3.50 – 3.45 (m, 2H), 3.17 (s, 9H), 2.12 (t, *J* = 7.7 Hz, 2H), 1.51 (p, *J* = 7.1 Hz, 2H), 1.26 (s, 16H), 0.85 (t, *J* = 6.8 Hz, 3H).


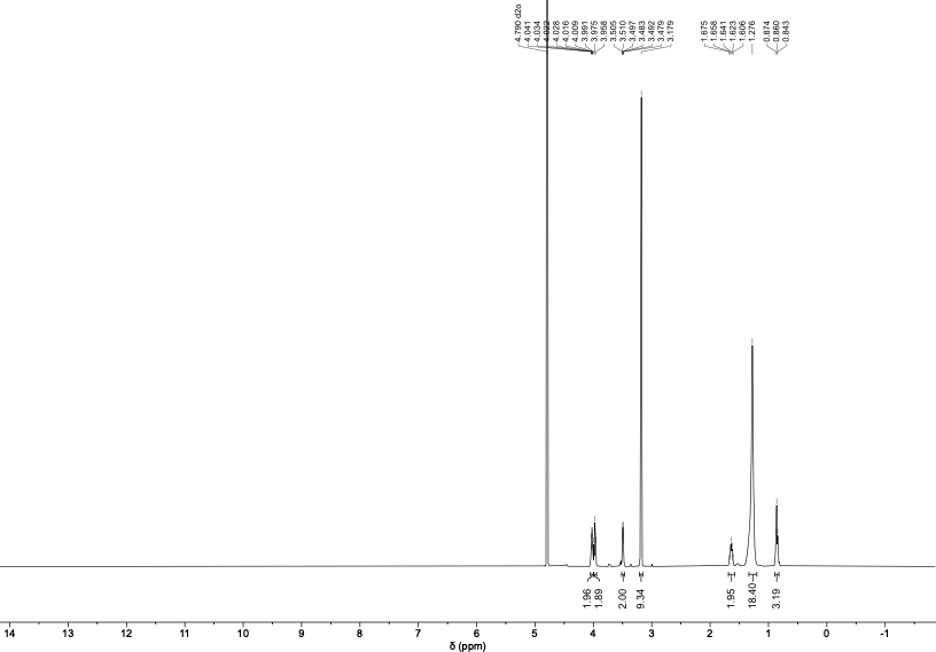


**Figure S19.** 1H NMR of choline dodecyl sulfate

1H NMR (400 MHz, D_2_O) δ 4.05 – 4.00 (m, 2H), 3.97 (t, J = 6.6 Hz, 2H), 3.52 – 3.46 (m, 2H), 3.18 (s, 9H), 1.64 (p, J = 6.7 Hz, 2H), 1.28 (s, 18H), 0.86 (t, J = 6.2 Hz, 3H).

**
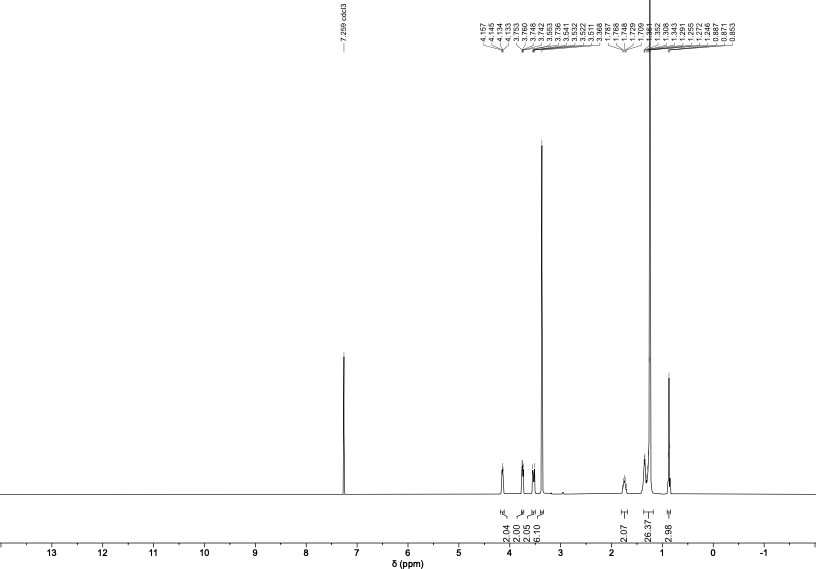
**

**Figure S20.** 1H NMR of surfactant-like choline bromide

^1^H NMR (400 MHz, CDCl_3_) δ 4.18 – 4.11 (m, 2H), 3.77 – 3.72 (m, 2H), 3.56 – 3.50 (m, 2H), 3.37 (s, 6H), 1.81 – 1.69 (m, 2H), 1.25 (s, 26H), 0.87 (t, *J* = 7.0 Hz, 3H).

**References**

1. Winther-Jensen O, Vijayaraghavan R, Sun J, Winther-Jensen B, MacFarlane DR. Self polymerising ionic liquid gel. Chemical Communications. 2009;3041–3.

2. Klein R, Kellermeier M, Touraud D, Müller E, Kunz W. Choline alkylsulfates - New promising green surfactants. J Colloid Interface Sci. 2013;392:274–80.

3. Mastellone G, Abbasi NM, Cagliero C, Anderson JL. New Class of Tunable Choline Bromide-Based Hydrophobic Deep Eutectic Solvents for the Extraction of Bioactive Compounds of Varying Polarity from a Plant Matrix. ACS Sustain Chem Eng. 2023;11:6665–75.

4. Kress WJ, Erickson DL, Jones FA, Swenson NG, Perez R, Sanjur O, et al. Plant DNA barcodes and a community phylogeny of a tropical forest dynamics plot in Panama. 2009.

5. Shioya N, Ogiso-Tanaka E, Watanabe M, Anai T, Hoshino T. Development of a High-Quality/Yield Long-Read Sequencing-Adaptable DNA Extraction Method for Crop Seeds. Plants. 2023;12:2971.

6. Kamiya M, Kiguchi T. Rapid DNA Extraction Method from Soybean Seeds. Breed Sci. 2003;53:277–9.

7. Espina MJC, Lovell JT, Jenkins J, Shu S, Sreedasyam A, Jordan BD, et al. Assembly, comparative analysis, and utilization of a single haplotype reference genome for soybean. The Plant Journal. 2024;120:1221–35.

8. Gel B, Serra E. karyoploteR: an R/Bioconductor package to plot customizable genomes displaying arbitrary data. Bioinformatics. 2017;33:3088–90.
